# Uncovering the distinct macro-scale anatomy of dysexecutive and behavioural degenerative diseases

**DOI:** 10.1093/brain/awad356

**Published:** 2023-10-13

**Authors:** Nick Corriveau-Lecavalier, Leland R Barnard, Hugo Botha, Jonathan Graff-Radford, Vijay K Ramanan, Jeyeon Lee, Ellen Dicks, Rosa Rademakers, Bradley F Boeve, Mary M Machulda, Julie A Fields, Dennis W Dickson, Neill Graff-Radford, David S Knopman, Val J Lowe, Ronald C Petersen, Clifford R Jack, David T Jones

**Affiliations:** Department of Neurology, Mayo Clinic, Rochester, MN 55905, USA; Department of Neurology, Mayo Clinic, Rochester, MN 55905, USA; Department of Neurology, Mayo Clinic, Rochester, MN 55905, USA; Department of Neurology, Mayo Clinic, Rochester, MN 55905, USA; Department of Neurology, Mayo Clinic, Rochester, MN 55905, USA; Department of Radiology, Mayo Clinic, Rochester, MN 55905, USA; Department of Neurology, Mayo Clinic, Rochester, MN 55905, USA; Department of Neuroscience, Mayo Clinic, Jacksonville, FL 32224, USA; Center for Molecular Neurology, Antwerp University, Antwerp, Belgium; Department of Neurology, Mayo Clinic, Rochester, MN 55905, USA; Department of Psychiatry and Psychology, Mayo Clinic, Rochester, MN 55905, USA; Department of Psychiatry and Psychology, Mayo Clinic, Rochester, MN 55905, USA; Department of Neuroscience, Mayo Clinic, Jacksonville, FL 32224, USA; Department of Neurology, Mayo Clinic, Jacksonville, FL 32224, USA; Department of Neurology, Mayo Clinic, Rochester, MN 55905, USA; Department of Radiology, Mayo Clinic, Rochester, MN 55905, USA; Department of Neurology, Mayo Clinic, Rochester, MN 55905, USA; Department of Radiology, Mayo Clinic, Rochester, MN 55905, USA; Department of Neurology, Mayo Clinic, Rochester, MN 55905, USA; Department of Radiology, Mayo Clinic, Rochester, MN 55905, USA

**Keywords:** dysexecutive Alzheimer’s disease, behavioural variant frontotemporal dementia, behavioural Alzheimer’s disease, FDG-PET, machine learning

## Abstract

There is a longstanding ambiguity regarding the clinical diagnosis of dementia syndromes predominantly targeting executive functions versus behaviour and personality. This is due to an incomplete understanding of the macro-scale anatomy underlying these symptomatologies, a partial overlap in clinical features and the fact that both phenotypes can emerge from the same pathology and vice versa. We collected data from a patient cohort of which 52 had dysexecutive Alzheimer’s disease, 30 had behavioural variant frontotemporal dementia (bvFTD), seven met clinical criteria for bvFTD but had Alzheimer’s disease pathology (behavioural Alzheimer’s disease) and 28 had amnestic Alzheimer’s disease. We first assessed group-wise differences in clinical and cognitive features and patterns of fluorodeoxyglucose (FDG) PET hypometabolism. We then performed a spectral decomposition of covariance between FDG-PET images to yield latent patterns of relative hypometabolism unbiased by diagnostic classification, which are referred to as ‘eigenbrains’. These eigenbrains were subsequently linked to clinical and cognitive data and meta-analytic topics from a large external database of neuroimaging studies reflecting a wide range of mental functions. Finally, we performed a data-driven exploratory linear discriminant analysis to perform eigenbrain-based multiclass diagnostic predictions. Dysexecutive Alzheimer’s disease and bvFTD patients were the youngest at symptom onset, followed by behavioural Alzheimer’s disease, then amnestic Alzheimer’s disease. Dysexecutive Alzheimer’s disease patients had worse cognitive performance on nearly all cognitive domains compared with other groups, except verbal fluency which was equally impaired in dysexecutive Alzheimer’s disease and bvFTD. Hypometabolism was observed in heteromodal cortices in dysexecutive Alzheimer’s disease, temporo-parietal areas in amnestic Alzheimer’s disease and frontotemporal areas in bvFTD and behavioural Alzheimer’s disease. The unbiased spectral decomposition analysis revealed that relative hypometabolism in heteromodal cortices was associated with worse dysexecutive symptomatology and a lower likelihood of presenting with behaviour/personality problems, whereas relative hypometabolism in frontotemporal areas was associated with a higher likelihood of presenting with behaviour/personality problems but did not correlate with most cognitive measures. The linear discriminant analysis yielded an accuracy of 82.1% in predicting diagnostic category and did not misclassify any dysexecutive Alzheimer’s disease patient for behavioural Alzheimer’s disease and vice versa. Our results strongly suggest a double dissociation in that distinct macro-scale underpinnings underlie predominant dysexecutive versus personality/behavioural symptomatology in dementia syndromes. This has important implications for the implementation of criteria to diagnose and distinguish these diseases and supports the use of data-driven techniques to inform the classification of neurodegenerative diseases.

## Teaser

Corriveau-Lecavalier *et al*. show that dementia syndromes targeting executive functions have different signatures of brain degeneration to those affecting personality and behaviour. This has implications for diagnosing and distinguishing these diseases, as well as for prognostic counselling and symptom management.

## Introduction

The diagnosis of neurodegenerative diseases progressively and predominantly targeting executive functions or behaviour/personality represents a challenge for clinicians and researchers, and there are many causes for this conundrum. This includes a partial overlap in clinical features including an early age at symptom onset, as generally observed in dysexecutive Alzheimer’s disease^1^ and behavioural variant frontotemporal dementia (bvFTD).^2,3^ There is also an incomplete understanding of the macro-scale anatomy underlying these symptomatologies, where both have traditionally been associated with frontal lobe dysfunction.^4-6^ For instance, early case studies have reported associations between isolated dysexecutive symptoms in the absence of prominent behavioural features in individuals who were found to have frontal lobe tau pathology at autopsy, suggesting the evidence of a so-called ‘frontal variant of Alzheimer’s disease’.^4,6^ However, recent evidence coming from neuroimaging studies suggests that executive functions would be subserved by more distributed patterns, notably involving lateral and medial parietal areas,^1,7-10^ echoing early electrophysiology studies in macaques revealing dense, reciprocal connections between dorsolateral prefrontal and posterior parietal cortices.^11,12^ Recent studies from our group on the early-onset dysexecutive phenotype of Alzheimer’s disease outline clinical brain-behaviour mappings consistent with this literature. Indeed, we have shown that the selective degeneration of the parieto-frontal network, and even parietal areas in isolation, in the setting of Alzheimer’s disease pathology resulted in a predominant progressive dysexecutive syndrome in the absence of behavioural features.^1,7,9,13^ On the other hand, predominant behavioural features in the context of degenerative dementia syndromes have consistently been associated with the degeneration of frontotemporal areas. More specifically, behavioural and personality changes have been associated with ventromedial prefrontal and orbitofrontal cortices as well as temporopolar areas with a right hemispheric predominance,^14-16^ which are heavily connected to the limbic system.^17^ It is, however, unclear whether behavioural features are inevitably accompanied by impairment of core executive functions, where studies have shown that social reasoning is far more impaired relative to other cognitive spheres in bvFTD compared with other degenerative phenotypes.^18-20^ An additional element of complexity pertains to the fact that the same underlying pathology can give rise to both phenotypes and vice versa. For instance, both Alzheimer’s and frontotemporal lobar degeneration pathologies can result in predominant dysexecutive and behavioural phenotypes,^21,22^ and around 20% of patients with a clinical diagnosis of bvFTD have Alzheimer’s pathology at autopsy,^23^ also known as ‘behavioural Alzheimer’s disease’. These factors collectively contribute to the high rate of misdiagnoses in early-onset dementia syndromes and the underestimation of their prevalence,^24^ and consequently undermine strategies to aid diagnosis, prognostic counselling, and symptom management and treatment.^3^

The combination of these elements has led the field to consider degenerative dysexecutive and behavioural syndromes along the same phenotypical spectrum rather than being distinct clinical entities. This is especially true in the setting of Alzheimer’s pathology,^25,26^ where these phenotypes have been conflated into the ‘behavioural or dysexecutive frontal variant’ of Alzheimer’s disease in various iterations of clinical criteria for the disease.^25,27^ There have been recent efforts to resolve this ambiguity. For instance, recently proposed diagnostic criteria for dysexecutive Alzheimer’s disease^1^ state that the dysexecutive syndrome must present in the absence of prominent behavioural features, i.e. not meeting criteria for bvFTD. A set of research criteria for behavioural Alzheimer’s disease have recently been proposed based on a systematic review and meta-analysis,^26^ which highlighted overlapping clinical features between behavioural Alzheimer’s disease and bvFTD. However, these criteria failed to reveal a specific imaging signature for behavioural Alzheimer’s disease due to the heterogeneity of the reviewed literature, and cognitive symptoms were often reported as the first clinical manifestation rather than behavioural problems. Moreover, no direct, data-driven comparisons of the recently defined dysexecutive predominant versus the established behavioural predominant dementia syndromes have been conducted.

In this study, we aimed to disambiguate the macro-scale anatomy of degenerative dementia syndromes predominantly targeting either executive functions or behaviour/personality, with the expectation that these phenotypes would have distinct signatures of network degeneration regardless of pathologic aetiology. We further supported these clinico-radiological associations by linking these patterns of degeneration to clinical and cognitive features. To this end, we leveraged group-wise comparisons and machine learning based on FDG-PET images of patients with dysexecutive Alzheimer’s disease, bvFTD, behavioural Alzheimer’s disease or amnestic Alzheimer’s disease. These analyses were conceptually grounded in the global functional state space, an information-processing model linking macro-scale anatomy, degeneration-related physiology and mental abilities,^28^ allowing the study of data-driven latent patterns of network degeneration unbiased by clinical classification.

## Materials and methods

### Participants

Patients were recruited from Mayo Clinic Rochester clinical practice with a subset enrolled in the Mayo Clinic Alzheimer’s Disease Research Center and/or the Advancing Research and Treatment in Frontotemporal Lobar Degeneration (ARTFL) Longitudinal Evaluation of Familial Frontotemporal Dementia Subjects (LEFFTDS) Longitudinal Frontotemporal Lobar Degeneration (ALLFTD) programme. Patients meeting criteria for dysexecutive Alzheimer’s disease presented with a predominant progressive dysexecutive syndrome in the absence of prominent behavioural features and had evidence of Alzheimer’s pathology.^1^ Given the relatively recent description of this dysexecutive Alzheimer’s disease, we provide a more detailed description of the diagnostic criteria used to diagnose this syndrome. A progressive dysexecutive syndrome was defined by presence of an insidious, continuous and persistent decline in mental functions, with the predominant feature being that of executive dysfunction. Meeting criteria for bvFTD is exclusionary, but having behavioural symptoms that do not meet bvFTD criteria or co-existing memory, language and/or visual impairment is not exclusionary if they originate from a predominant impairment in any core executive function (i.e. working memory, cognitive flexibility and/or inhibition). Construct validity for defining this clinical syndrome in this way was demonstrated in the original report^1^ and recently via mapping of this clinical syndrome to brain anatomy associated with executive function as opposed to memory, visual or language functions as is the case for other Alzheimer’s disease associated clinical syndromes predominantly affecting those domains.^28^ All patients with dysexecutive Alzheimer’s disease were part of a previous publication from our group characterizing the clinico-radiological heterogeneity of dysexecutive Alzheimer’s disease,^7^ and 46/52 were part of the initial report defining this syndrome.^1^

Patients meeting criteria for bvFTD presented with a predominant behavioural syndrome in line with Rascovsky criteria^2^ (three of the following: early behavioural disinhibition, early apathy or inertia, early loss of sympathy or empathy, early perseverative, stereotyped or compulsive behaviour, hyperorality or dietary changes, executive dysfunction). Nine of these patients additionally met criteria for definite bvFTD either because they carried a genetic mutation (*C9orf72*, *GRN*, *MAPT*) or had autopsy-proven pathology associated with frontotemporal lobar degeneration. Patients with behavioural Alzheimer’s disease met clinical criteria for bvFTD but were found to have evidence for Alzheimer’s pathology. Patients meeting criteria for amnestic Alzheimer’s disease presented with a predominant amnestic syndrome and had evidence for Alzheimer’s pathology.^29^ Alzheimer’s disease biomarkers thresholds (CSF, PET) and procedures for genetic testing and post-mortem assessment are described later. The *APOE* genotype was available for 84/117 patients.

All diagnoses occurred in the behavioural neurology practice in Rochester, MN, USA using clinical standards adopted by experts in the field. Clinical assessments were performed with the patient and an informant by experienced neurologists subspecialized in behavioural neurology. They were conducted through a structured interview covering various cognitive and behavioural symptoms related to the core clinical criteria for the clinical syndromes included in this study. Additional diagnostic assessment occurred for participants co-enrolled in research programmes using structured instruments including structured interviews with patients and co-participants with consensus panel review of all diagnoses in line with diagnostic standards in the field and standard protocols of National Institute on Aging-funded Alzheimer’s Disease Centers programmes.^30,31^ Fluorodeoxyglucose (FDG)-PET images were used directly for diagnostic purposes in 48/117 patients (e.g. supporting clinical impressions, differential diagnosis, objectively quantifying severity and evaluating for co-pathology). In the remaining cases, FDG-PET was ordered after they received their clinical diagnosis, either in clinical or research settings. Of note, most FDG-PET scans used in the context of this study were acquired in research settings, following the initial scan used for diagnostic purposes in clinical practice (total of 92/117 FDG-PET scans).

Data from 117 age- and sex-matched cognitively unimpaired controls from the Mayo Clinic Study of Aging with available FDG-PET and amyloid-PET were collected for comparison purposes. All control participants had to be amyloid-negative based on PET imaging to be included in the study, and those with available tau-PET also had to be tau-negative.

Patients and/or their legal representative provided written informed consent for their data to be used for research purposes. This study met Health Insurance Portability and Accountability Act (HIPAA) guidelines and was approved by the Mayo Clinic Institutional Review Board.

### CSF biomarkers

CSF Alzheimer’s disease biomarkers were collected in 25/52 dysexecutive Alzheimer’s disease, 5/30 bvFTD, 3/7 behavioural Alzheimer’s disease and 3/28 amnestic Alzheimer’s disease patients. CSF assessment was performed by Athena Diagnostic (Worcester, MA, USA). Amyloid-β (Aβ)_42_, t-tau and p-tau levels were assessed in addition to an Aβ_42_/p-tau index (ATI). Cut-offs for Alzheimer’s pathology were: (i) not consistent with Alzheimer’s disease (p-tau < 54 pg/ml; ATI > 1.2); (ii) borderline Alzheimer’s disease (p-tau 54–58 pg/ml; ATI 0.8–1.2); or (iii) consistent with Alzheimer’s disease (p-tau > 58 pg/ml; ATI < 0.8).

### Post-mortem assessment

Post-mortem assessment was used to confirm Alzheimer’s disease pathology in 1/52 dysexecutive Alzheimer’s disease and 4/7 behavioural Alzheimer’s disease patients, and frontotemporal lobar degeneration (i.e. 4R tauopathies, TDP-43) pathology in 3/30 bvFTD patients. All neuropathological assessments were performed by an experienced neuropathologist in accordance with current diagnostic protocols.^32^ Immunochemistry was performed using a battery of antibodies for α-synuclein (rabbit polyclonal; NACP, Mayo Clinic antibody, 1:3000 with 95% formic acid pretreatment and DAKO EnVision reagents), phosphorylated TDP-43 antibody (pS409/410, 1:5000 mouse monoclonal, Cosmo Bio Co. Ltd.), Aβ (6F/3D, 1:250, human Aβ_8–17_, DAKO), p-tau (CP13; 1:1000; IgG1 to phosphoserine 202), 4R-tau (RD4, 1:5000, Millipore) and 3R-tau (RD3, 1:5000, Millipore). Senile plaques and neurofibrillary tangles were assessed with thioflavin S fluorescent microscopy and Bielschowsky and Gallyas silver stains, respectively.

Pathological diagnoses were assigned according to published criteria. Alzheimer’s disease was diagnosed according to the ABC ranking score,^33^ which includes the Thal staging of amyloid plaques, Braak staging of neurofibrillary tangles (0–VI)^34^ and the density measurement of neuritic plaques (0–3).^32^ Corticobasal basal degeneration (4R tauopathy) was diagnosed by the presence of cortical and subcortical neuronal and glial lesions (i.e. astrocytic plaques) and thread-like processes in grey and white matter.^35^ Lewy body disease (LBD) was staged according to published criteria.^36^ TDP-43 type A was defined as TDP-43 immunoreactive neuronal cytoplasmic inclusions, dystrophic neurites and neuronal intranuclear inclusions in vulnerable cortical and subcortical areas. TDP-43 type B had predominantly neuronal cytoplasmic inclusions.^37^ TDP-43 staging was classified as FTLD-related or non-FTLD-related. In the latter case, TDP-43 staging was done according to the limbic age-related TDP-43 encephalopathy (LATE) staging.^38^ The presence of other pathologies such as hippocampal sclerosis (regardless of TDP-43 involvement), and vascular disease (cerebral amyloid angiopathy, microinfarcts and large, lacunar and haemorrhagic infarcts) were also assessed.^36^

### Genetic testing

The presence of frontotemporal lobar degeneration-related genetic mutations was assessed and confirmed in 7/30 bvFTD. DNA samples were analysed at the University of California, Los Angeles in the context of ALLFTD participation using published methods.^39^ Samples were screened using targeted sequencing of a custom panel of genes previously demonstrated to be implicated in frontotemporal lobar degeneration, including *MAPT* and *GRN*. The presence of hexanucleotide repeat expansions in *C9orf72* was assessed using both fluorescent and repeat-primed PCR.

### Amyloid-PET and tau-PET imaging

Amyloid-PET was acquired in 26/52 dysexecutive Alzheimer’s disease, 1/7 behavioural Alzheimer’s disease and all amnestic Alzheimer’s disease patients and controls. Tau-PET was acquired in 18/52 dysexecutive Alzheimer’s disease, 1/7 behavioural Alzheimer’s disease, 22/28 amnestic Alzheimer’s disease patients and 50/117 controls. Amyloid-PET and tau-PET images were acquired using Pittsburgh compound (PiB) and ^18^F-AV-1451 (AV1451) ligands, respectively. Image processing and abnormality cut-offs are described in separate publications.^40,41^ Briefly, images were normalized to the cerebellar crus region and a global standardized uptake value ratio (SUVR) was computed from a validated meta-region of interest for each participant. Abnormality SUVR cut-offs for amyloid-PET and tau-PET were set at >1.42 and >1.29, respectively. Images were co-registered to their respective T1-weighted MRI, normalized to the Mayo Clinic Aging and Lifespan Template (MCALT) and smoothed with a 6-mm full-width at half-maximum (FWHM) kernel.

### FDG-PET imaging

FDG-PET was acquired for all participants using a PET/CT scanner (General Electronics Healthcare) after a 30-min uptake period in a dimly lit room. The duration of the scanning session was 8 min, which was split into four 2-min dynamic frames following a low-dose CT transmission scan. Images were processed using an MRI-free pipeline for which steps included registration to the MCALT space using non-linear symmetric diffeomorphic registration, standardization of the FDG-PET signal to the pons and smoothing using a 6-mm FWHM kernel.

### Cognitive assessment

All patients were administered the Short Test of Mental Status (STMS),^42^ which is a bedside cognitive screening test reflecting global cognitive function. Neuropsychological assessment was performed in 35/52 dysexecutive Alzheimer’s disease, 23/30 bvFTD, 4/7 behavioural Alzheimer’s disease and 24/28 amnestic Alzheimer’s disease patients and covered a variety of cognitive domains, including: cognitive flexibility, inhibition, verbal and visual working memory, verbal and visual episodic memory, verbal fluency, visuoconstruction and visuospatial reasoning (Table 1 for the list of tests). Test selection was not standardized and varied as a function of the setting in which it was performed (i.e. clinical versus research) and/or degree of cognitive impairment. Composite scores were computed by averaging scaled scores of tests included in each domain, except for the Wisconsin Card Sorting Test (WCST) and Trail Making Test B (TMT-B), which were considered separately. Raw scores were transformed into age-adjusted scaled scores according to respective manuals for the Wechsler Adult Intelligence Scale (WAIS), Wechsler Memory Scale (WMS) and Delis–Kaplan Executive Function System (D-KEFS), and Mayo Older Americans Normative Studies^43-46^ for the remaining tests.

**Table 1:** Cognitive and neuropsychological tests

Cognitive domain	Test
Bedside cognitive screening	STMS^42^
Cognitive flexibility	TMT-B^47^
	WCST; perseverative errors^48^
Inhibition	Stroop test: Inhibition condition^49^
Verbal working memory	WAIS-III/WAIS-IV: digit span, arithmetic, letter-number sequencing^50,51^
	WMS-III: mental control^52^
Visuospatial working memory	WMS-III: spatial span^52^
Verbal episodic memory	RAVLT^53^
Visual episodic memory	WMS-III Visual Reproduction I and II^52^
Verbal fluency	Animal and phonemic fluency^54^
Visuoconstruction	WAIS-III/WAIS-IV: block design^50,51^
	ROCF copy^55^
Visuospatial reasoning	WAIS-III/WAIS-IV: picture completion, matrix reasoning, visual puzzles^50,51^

RAVLT = Rey Auditory Verbal Learning Test; ROCF = Rey–Osterrieth Complex Figure; STMS = Short Test of Mental Status; TMT-B = Trail Making Test-B; WAIS = Weschler Adult Intelligence Scale; WCST = Wisconsin Card Sorting Test; WMS = Weschler Memory Scale.

### Spectral covariance decomposition analysis

We used an analytic framework called ‘biological projection and reduction’^7,28,56^ to yield unsupervised, data-driven and biologically interpretable latent variables based on FDG-PET images that parametrizes interindividual variability in patterns of hypometabolism across the patient cohort being investigated. This was done in Python version 3.7.12 using libraries developed in-house. This first involved the median-centring and interquartile scaling of individual pons-normalized FDG-PET images censored by a brain tissue mask. These images were then entered into a participant-by-voxel matrix. This matrix was then submitted to a singular value decomposition to derive a set of data-driven latent factors, or ‘eigenbrains’, which are independent from clinical diagnostic labels. These eigenbrains are represented by patterns of metabolism organized along dimensions determined by their spatial distribution and magnitude of intensity. It is important to bear in mind that an eigenbrain does not reflect hypometabolism *per se*, but rather a relative distribution of metabolism across the entire brain with opposing poles of relative hypo- and hyper-metabolism. Here, a patient could express less metabolism in a set of regions from one pole relative to the other pole, and another patient could show the opposite pattern. This directionality is determined by an ‘eigenvalue’, which can be either positive or negative and describes how the pattern of metabolism at the patient-level relates to the topology and directionality of the eigenbrain. In other words, eigenbrains are latent factors representative of relative metabolism at the cohort-level, whereas eigenvalues are values at the patient-level reflecting how a given participant is represented by a given eigenbrain. It is noteworthy that the eigenbrains generated by the singular value decomposition are statistically orthogonal. This means that the eigenvalues on each eigenbrain are totally independent within a single patient. In other words, the fact that two eigenbrains share topological overlap does not mean that a patient will load similarly on both eigenbrains. Individual eigenvalues can be subsequently linked to clinical/cognitive data. The number of significant eigenbrains was determined using Horn’s method,^57^ which proposes that factoring should cease when factors cannot account for a proportion of variance that is higher than expected by chance. The amount of covariance explained by a given eigenbrain is expressed as the percentage of absolute variance across FDG-PET images of the whole cohort.

### Meta-analytic decoding

We performed a meta-analytic decoding of each eigenbrain using the Neurosynth database (https://neurosynth.org/)^58,59^ with the 50 topics list (https://neurosynth.org/analyses/topics/). Consistent with other studies using this approach,^7,28,60,61^ we retained only topics capturing coherent mental functions for a total of 22 topics. This procedure involves the assessment of topics that best align with a given eigenbrain, allowing for an interpretation of brain-behaviour relationships leveraging a large external database of functional neuroimaging studies. Of note, interpretation should be guided by the directionality and relative strength of associations between eigenbrains and meta-analytic topics rather than absolute coefficient values for reasons cited on the Neurosynth website (https://neurosynth.org/faq/#q16). It is important to keep in mind that this analysis is meant to be descriptive, and no statistical significance testing was performed.

### Statistical analyses

Statistical analyses were performed using R version 4.2.3. Demographic, clinical and cognitive differences across patient groups were assessed with ANOVAs for continuous variables and Tukey’s test for *post hoc* comparisons when the omnibus test was significant, and chi-squared analysis for categorical variables.

Between-group differences in patterns of FDG-PET hypometabolism were assessed by fitting the mean image of a given patient group to the mean image a group of cognitively unimpaired subjects matched for group size, age and sex, resulting in pair-wise *Z*-score maps. Group-wise mean images were scaled by their respective standard deviation at the voxel-level. These comparisons were also computed between patient groups (e.g. dysexecutive Alzheimer’s disease versus bvFTD, etc.).

Eigenvalues were compared across each patient group for each eigenbrain using ANOVAs and Tukey’s test for *post hoc* comparisons. This was done to assess how the patterns expressed by these eigenbrains differed across patient groups. Associations between eigenvalues and demographic, cognitive, behavioural and *APOE* data were assessed using multivariable linear and logistic regression frameworks where eigenvalues of each eigenbrain were entered as predictors of the variable of interest. These associations were thus expressed as standardized *β* coefficients for continuous variables and odds ratios for categorical variables.

Finally, we performed a linear discriminant analysis with eigenvalues of the significant eigenbrains as input features to perform data-driven multiclass diagnostic predictions. This supervised machine learning technique aims to find a linear combination of features that best discriminates between different classes (i.e. diagnostic category) by projecting a high-dimensional feature space (i.e. eigenvalues) onto a lower-dimensional subspace, while preserving the class-discriminatory information as much as possible. The combination of these linear discriminants was used to predict the clinical diagnosis of each patient based on the input features, and these predictions where then compared with true clinical diagnosis. We performed a series of Levene’s tests to assess the presence of heteroscedasticity in input features, i.e. eigenvalues, according to group. This revealed slight yet significant variance inhomogeneity in eigenvalues. Thus, we assessed the potential influence of heteroscedasticity on the results by performing: (i) a linear discriminant analysis using log-transformed eigenvalues as input features; and (ii) a robust linear discriminant analysis designed to account for non-normal distributions. We additionally assessed the associations between the linear discriminants yielded by this analysis and age using simple regression models and mapped sex onto the low-dimensional space to assess the potential influence of these variables on our results.

## Results

### Demographic, biomarker, *APOE* and clinical/cognitive comparisons

The results are summarized in Table 2. Dysexecutive Alzheimer’s disease and bvFTD patients were younger at symptom onset, followed by behavioural Alzheimer’s disease, then amnestic Alzheimer’s disease. All dysexecutive, behavioural and amnestic Alzheimer’s disease patients with available CSF and/or PET data were classified as amyloid- and tau-positive, and those who underwent autopsy had Alzheimer’s disease as their primary pathological diagnosis. bvFTD patients with available CSF biomarkers had an ATI inconsistent with Alzheimer’s disease, and those who underwent autopsy examination had frontotemporal lobar degeneration as their primary pathological diagnosis. Complete neuropathological findings are listed in Supplementary Table 1. All cognitively unimpaired participants were amyloid-negative, and those with available tau-PET were tau-negative. There were significantly more *APOE4* carriers in the amnestic Alzheimer’s disease group compared with the bvFTD group.

**Table 2:** Demographic, cognitive, Alzheimer’s disease biomarker and *APOE* genotype data

	dAD (*n* = 52)	bvFTD (*n* = 30)	bvAD (*n* = 7)	aAD (*n* = 28)	*P*-value
**Demographic and clinical**					
Age at symptom onset	53.70 (5.35)	54.40 (11.20)	62.70 (5.53)	74.90 (4.83)	<0.001^a^
Age at FDG-PET scan	57.10 (5.30)	59.2 (9.97)	66.40 (5.38)	78.6 (4.33)	<0.001^a^
Disease duration: age at symptom onset, *n* = 33	8.67 (2.61)	6.67 (3.20)	7.75 (4.50)	8.33 (1.80)	0.55
Disease duration: age at FDG, *n* = 33	5.17 (3.1)	3.67 (1.75)	4.5 (1.29)	4.09 (1.92)	0.41
Males, females	34, 18	13, 17	4, 3	16, 12	0.3
Education	15.10 (2.22)	15.30 (2.35)	17.00 (2.10)	15.20 (3.22)	0.4
STMS	21.20 (8.59)	29.40 (6.05)	30.00 (5.20)	27.10 (4.44)	<0.001^b^
Social disinhibition	0/52 (0d)	25/30 (83%)	5/7 (71%)	1/28 (3%)	<0.001^c^
Apathy	14/52 (27%)	19/30 (63%)	4/7 (57%)	2/28 (7%)	<0.001^d^
Lack of empathy	0/52 (0%)	9/30 (30%)	3/7 (43%)	0/28 (0%)	<0.001^c^
Perseverative/obsessive behaviour	0/52 (0%)	10/30 (30%)	6/7 (86%)	0/28 (0%)	<0.001^c^
Hyperorality/dietary habit changes	1/52 (2%)	15/30 (50%)	3/7 (43%)	0/28 (0%)	<0.001^c^
Executive dysfunction	52/52 (100%)	30/30 (100%)	7/7 (100%)	22/28 (79%)	<0.001^e^
**Cognitive performance [*n* per group]**					
Working memory, *n* = 52	5.73 (2.64) [30]	8.39 (2.98) [14]	8.67 (2.89) [3]	10.5 (2.55) [5]	<0.001^b^
Inhibition, *n* = 65	3.72 (2.84) [24]	6.55 (3.61) [22]	N/A (0)	7.17 (3.11) [18]	0.0012^b^
Trail Making Test-B, *n* = 87	2.62 (2.62) [32]	5.54 (4.52) [28]	5.25 (6.13) [4]	5.48 (3.98) [23]	0.014^b^
WCST perseverative errors, *n* = 16	5.7 (2.36) [10)	9.2 (1.10) [5]	9^f^ (1)	N/A (0)	0.02^g^
Verbal fluency, *n* = 91	5.76 (3.22) [35]	5.55 (2.64) [28]	5.88 (3.47) [4]	8.02 (3.16) [24]	0.019^h^
Visuoconstruction, *n* = 79	4.64 (2.66) [32]	8.8 (3.24) [23]	4.33 (2.52) [3]	8.75 (3.46) [20]	<0.001^b^
Visuospatial, *n* = 43	6.25 (2.49) [24]	10.3 (2.5) [13]	11^f^ (1)	10.5 (2.29) [5]	<0.001^b^
Verbal episodic memory—immediate recall, *n* = 75	4.11 (2.16) [33]	6.5 (2.41) [19]	5.17 (3.21) [3]	7.82 (2.48) [20]	<0.001^b^
Verbal episodic memory—delayed recall, *n* = 75	3.41 (1.85) [33]	5.39 (3.15) [19]	3 (1.1.5) (3)	4.97 (1.44) [20]	0.0059^g^
Visual episodic memory—immediate recall, *n* = 33	2.91 (1.9) [23]	6.86 (1.68) [7]	6^f^ (1)	7 (0.00) [2]	<0.001^b^
Visual episodic memory—delayed recall, *n* = 33	4.25 (2.31) [23]	6.14 (2.27) [7]	9^f^ (1)	2 (0.00) [2]	0.06
**Alzheimer’s disease biomarkers and *APOE4***					
Amyloid-PET availability	26	N/A	1	28	–
Tau-PET availability	18	N/A	1	22	–
CSF availability	42	5	3	3	–
Autopsy data availability	1	3	4	7	–
*APOE4* data availability	29	28	1	26	–
CSF Aβ_42,_*n* = 52	359 (283–477)	1084 (984–1079)	612 (601–706)	397 (280–476)	–
CSF p-tau, *n* = 52	76.3 (59.8–92.4)	31.1 (2.2–46.5)	27.4 (25.9–40.3)	80.6 (59.6–94.00)	–
CSF t-tau, *n* = 52	504 (354–838)	149 (66–353)	271 (264–373)	632 (452–664)	–
ATI, *n* = 52	0.42 (0.26–0.58)	2 (2–319)	0.41 (0.32–0.43)	0.40 (0.34–0.46)	–
PiB SUVR, *n* = 55	2.39 (2.26–2.59)	N/A	2.15**^f^**	2.60 (2.38–2.80	–
AV1451 SUVR, *n* = 41	2.1 (1.9–2.6)	N/A	1.29**^f^**	1.73 (1.43–1.90)	–
*APOE4* carriers (%), *n* = 84	15/29 (52)	6/28 (21)	0/1 (0)	18/26 (69)	0.003^i^

Cognitive scores are expressed as age-adjusted scaled scores, with mean and standard deviation in parentheses. Biomarker data are expressed as median (interquartile range). Aβ = amyloid-beta; AD = Alzheimer’s disease; aAD = amnestic AD; dAD = dysexecutive AD; ATI = amyloid/tau index; AV1451 = ^18^F-AV-1451; bvFTD = behavioural variant frontotemporal dementia; bvAD = behavioural AD; FDG = fluorodeoxyglucose; N/A = not available; PiB = Pittsburgh compound; STMS = Short Test Of Mental Status; WCST = Wisconsin Card Sorting Test; SUVR = standardized uptake value ratio.

^a^dAD and bvFTD < bvAD < aAD.

^b^dAD < bvFTD and aAD.

^c^dAD and aAD < bvAD and bvFTD.

^d^aAD < bvAD and bvFTD; dAD < bvFTD.

^e^aAD < dAD and bvFTD and bvAD.

^f^Only one participant.

^g^dAD < bvFTD.

^h^dAD and bvFTD < aAD.

^i^aAD > bvFTD.

Comparisons of behavioural features revealed that bvFTD and behavioural Alzheimer’s disease patients were more likely to present with social disinhibition, lack of empathy and hyperorality compared with dysexecutive and amnestic Alzheimer’s disease, and more likely to manifest apathy compared with amnestic Alzheimer’s disease. Comparisons of cognitive measures showed that dysexecutive Alzheimer’s disease patients were significantly more impaired than bvFTD and amnestic Alzheimer’s disease patients on the STMS and nearly all cognitive measures. However, dysexecutive Alzheimer’s disease and bvFTD patients both had significantly lower verbal fluency performance compared with amnestic Alzheimer’s disease patients, and dysexecutive Alzheimer’s disease patients had worse performance on the WCST and delayed verbal episodic memory recall compared with bvFTD only. There was no significant difference between behavioural Alzheimer’s disease patients and all three other groups on cognitive measures. Disease duration as calculated by the time between age at reported symptom onset and age at death or between age at FDG and age at death did not significantly differ between phenotypic groups.

### FDG-PET group-wise comparisons

**Figure 1:**
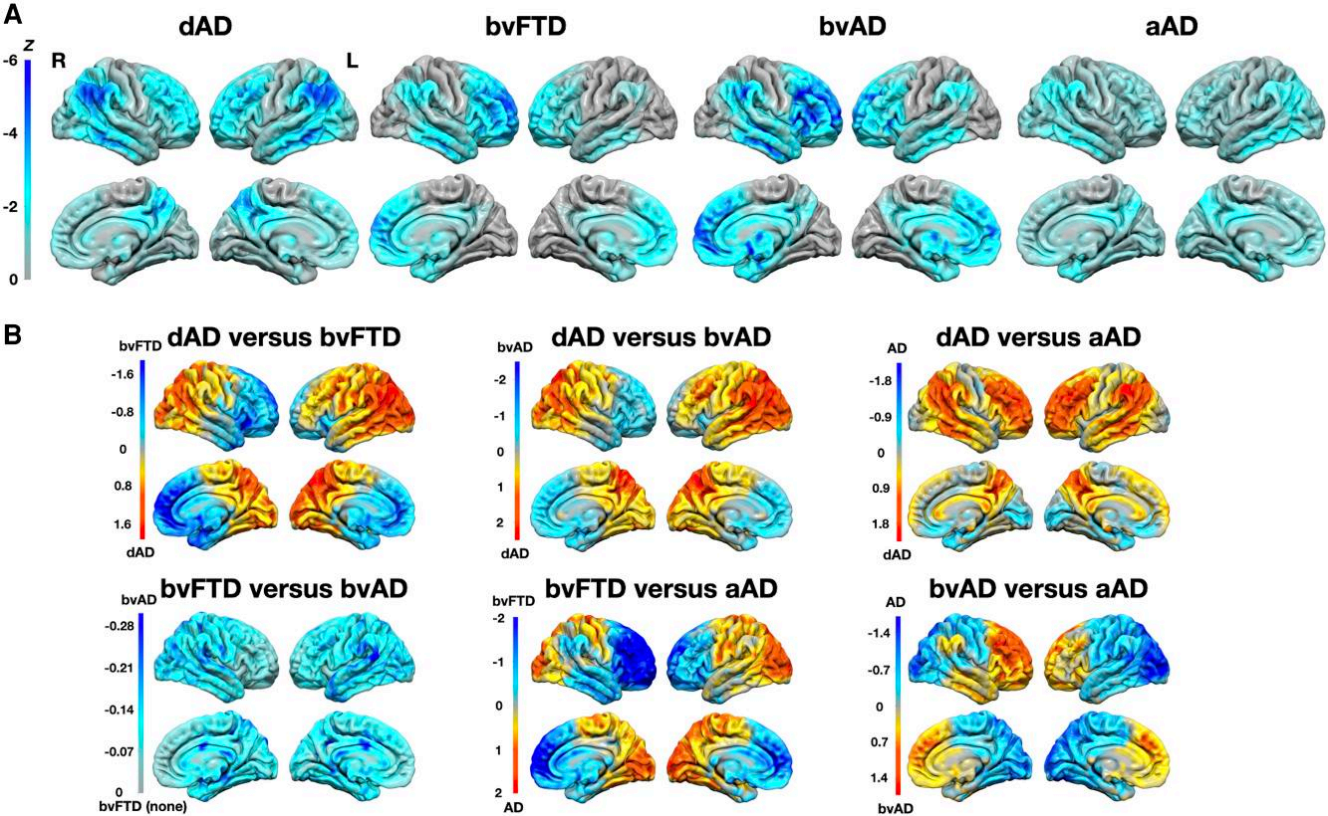
**Group-wise comparisons of FDG-PET hypometabolism**. (**A**) Pair-wise comparisons between each patient group and cognitively unimpaired controls matched for sample size, age and sex. (**B**) Pair-wise comparisons between patient groups, where colour codes oppose patterns of hypometabolism relative to each patient group included in the comparison. Differences are expressed in *Z*-score at the voxel-level. aAD = amnestic Alzheimer’s disease; bvAD = behavioural Alzheimer’s disease; bvFTD = behavioural variant of frontotemporal dementia; dAD = dysexecutive Alzheimer’s disease; FDG = fluorodeoxyglucose; L = left; R = right.

FDG-PET comparisons are displayed in Fig. 1. Compared with controls, dysexecutive Alzheimer’s disease patients exhibited hypometabolism in heteromodal cortices bilaterally. Behavioural Alzheimer’s disease and bvFTD patients showed similar patterns of hypometabolism compared with their controls counterparts, involving mostly prefrontal areas, and to a lesser extent inferior temporal and lateral parietal areas with a right hemispheric predominance. Amnestic Alzheimer’s disease patients had a milder pattern of hypometabolism most prominent in temporo-parietal areas and frontal areas bilaterally although to a lesser extent.

Comparisons between patient groups revealed that dysexecutive and amnestic Alzheimer’s disease patients had greater parieto-occipital hypometabolism compared with behavioural Alzheimer’s disease and bvFTD patients. Dysexecutive Alzheimer’s disease additionally had greater hypometabolism of left prefrontal dorsolateral areas compared with behavioural Alzheimer’s and bvFTD. Behavioural Alzheimer’s disease and bvFTD had greater involvement of right temporo-parietal areas compared with amnestic Alzheimer’s disease. Dysexecutive Alzheimer’s disease patients had greater hypometabolism in heteromodal cortices compared with amnestic Alzheimer’s disease patients, whereas amnestic Alzheimer’s disease patients had greater hypometabolism in medial temporal, orbitofrontal and occipital regions. There was no meaningful difference between bvFTD and behavioural Alzheimer’s disease patients, with *Z*-scores not exceeding 0.3 at the voxel-level.

### Spectral covariance decomposition of FDG-PET

**Figure 2:**
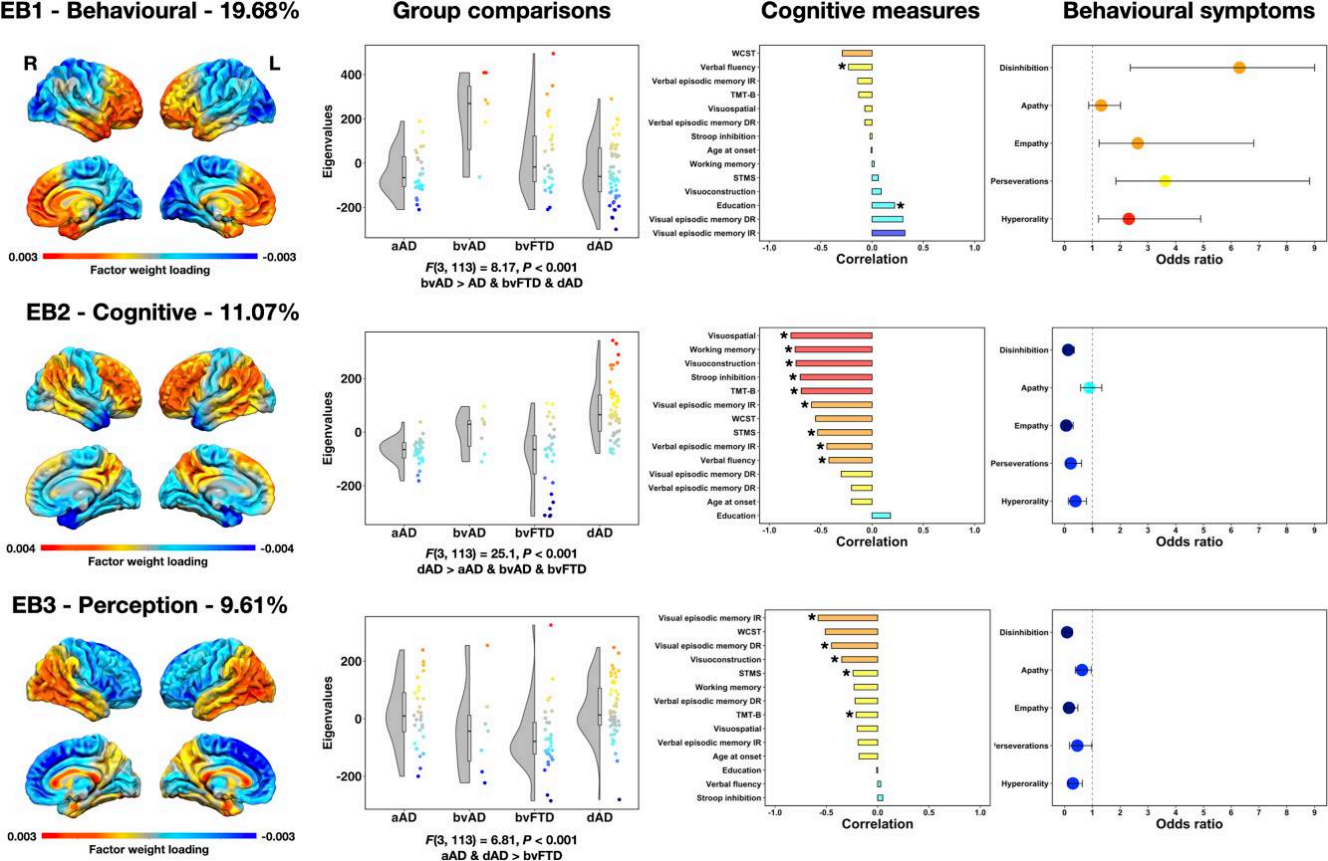
**Associations between eigenbrains, diagnostic group, cognition and behavioural/personality symptoms**. The colour code indicates areas of relative FDG-PET hypometabolism highlighted by a given eigenbrain, and their relationships with diagnostic membership, cognition, behavioural/personality symptoms. These eigenbrains reflect relative metabolism between two sets of brain areas, and the directionality (positive or negative) is arbitrary. The percentage of covariance explained between FDG-PET images is displayed above each eigenbrain rendering. Asterisks in the bar plots for cognitive measures indicate significant correlations. Odds ratios for behavioural symptoms are significant if the confidence intervals do not overlap with 1, where small values indicate a lower likelihood and higher values a higher likelihood. aAD = amnestic Alzheimer’s disease; bvAD = behavioural Alzheimer’s disease; bvFTD = behavioural variant of frontotemporal dementia; dAD = dysexecutive Alzheimer’s disease; DR = delayed recall; EB = eigenbrain; IR = immediate recall; L = left; R = right; STMS = Short Test of Mental Status; WCST = Wisconsin Card Sorting Test.

**Figure 3:**
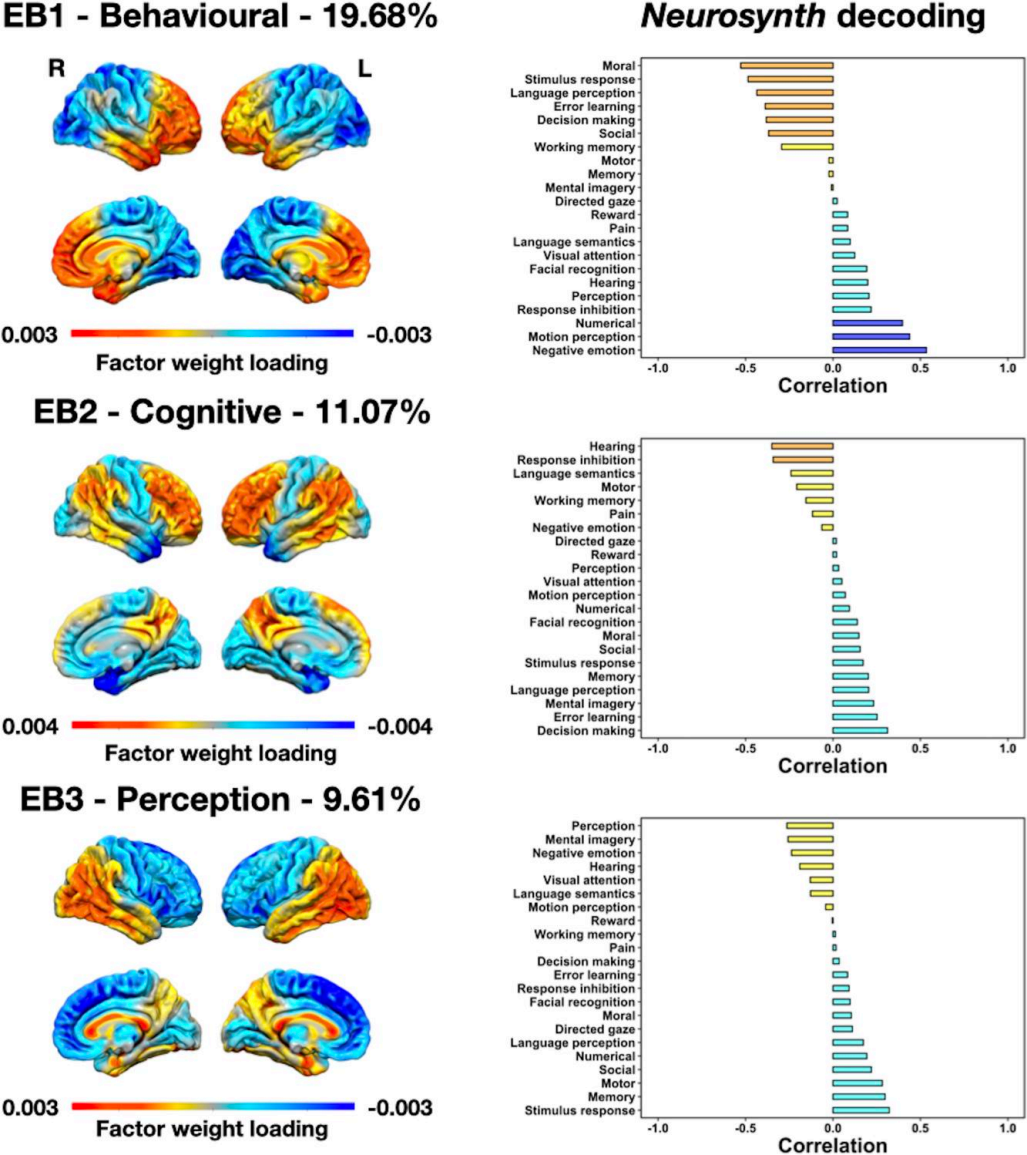
**Associations between and meta-analytic Neurosynth topics**. The colour code indicates areas of relative FDG-PET hypometabolism highlighted by a given eigenbrain (EB), and their relationships with meta-analytic topics. These EBs reflect relative metabolism between two sets of brain areas, and the directionality (positive or negative) is arbitrary. The percentage of covariance explained between FDG-PET images is displayed above each EB rendering. Relationships should be interpreted according to their direction and relative magnitude rather than absolute coefficients. FDG = fluorodeoxyglucose; L = left; R = right.

There were nine significant eigenbrains in total. Only eigenbrains 1–3 are presented in the main text for the sake of concision and clarity, and because they explained the highest proportion of covariance in FDG-PET images across the whole cohort. Eigenbrains 4–9 can be found in Supplementary Fig. 1. It is, however, of note that all eigenbrains were used for the linear discriminant analysis described below. Eigenbrains 1–3 and their relationships with phenotypic differences, cognitive domains and behavioural symptoms are displayed in Fig. 2, and their relationships with Neurosynth topics are displayed in Fig. 3. Scatterplots between eigenvalues and demographic and cognitive scores for eigenbrains 1–3 can be found in Supplementary Figs 2–4. Table 3 lists the relationships between all nine eigenbrains and demographic, clinical, cognitive and *APOE4* variables. Supplementary Table 2 summarizes Neurosynth decoding results for all eigenbrains.

**Table 3:** Relationships between eigenbrains and demographic, clinical and cognitive data

Variable	*R^2^*	$R_{\mathrm{adj}}^{2}$	EB1	EB2	EB3	EB4	EB5	EB6	EB7	EB8	EB9	*P*-value
**Continuous variables (expressed as beta coefficients)**												
Age at symptom onset	0.39	0.34	0.01	**−0**.**20**	−0.18	0.11	0.09	**−0**.**20**	**0**.**42**	−0.05	**−0**.**28**	**<0**.**001**
Education	0.08	0.00	**−0**.**22**	0.18	−0.01	−0.02	0.07	−0.03	0.06	−0.06	0.01	0.495
STMS	0.51	0.47	−0.06	**−0**.**53**	**−0**.**24**	**0**.**25**	**0**.**37**	−0.10	−0.04	−0.04	−0.08	**<0**.**001**
Working memory	0.55	0.45	−0.02	**−0**.**75**	−0.23	0.02	0.08	−0.07	−0.05	−0.16	0.01	**<0**.**001**
Inhibition	0.50	0.42	0.02	**−0**.**70**	0.05	0.20	0.02	0.17	0.04	**−0**.**25**	−0.03	**<0**.**001**
Trail Making Test B	0.41	0.34	0.13	**−0**.**69**	**−0**.**21**	0.06	0.10	0.04	−0.14	−0.14	−0.08	**<0**.**001**
WCST perseverative errors	0.72	0.29	0.29	−0.55	−0.51	0.25	−0.09	−0.31	0.46	0.01	−0.22	0.272
Verbal fluency	0.32	0.25	**0**.**23**	**−0**.**42**	0.03	0.02	**0**.**33**	−0.06	−0.09	−0.08	−0.08	**<0**.**001**
Visuoconstruction	0.63	0.58	−0.09	**−0**.**74**	**−0**.**35**	**0**.**29**	**−0**.**20**	**−0**.**18**	0.07	**−0**.**26**	−0.10	**<0**.**001**
Visuospatial	0.70	0.06	0.07	**−0**.**79**	−0.20	**0**.**34**	**−0**.**27**	−0.01	0.12	**−0**.**31**	−0.15	**<0**.**001**
Verbal episodic memory IR	0.37	0.28	0.14	**−0**.**44**	−0.19	0.07	0.17	−0.10	**0**.**25**	0.07	−0.14	**<0**.**001**
Verbal episodic memory DR	0.22	0.11	0.07	−0.20	−0.22	0.01	0.20	−0.01	**0**.**32**	0.02	0.02	**0**.**049**
Visual episodic memory IR	0.59	0.44	−0.32	**−0**.**59**	**−0**.**58**	0.18	0.14	−0.11	0.14	−0.05	−0.05	**0**.**005**
Visual episodic memory DR	0.38	0.15	−0.30	−0.30	**−0**.**45**	0.12	0.24	−0.04	−0.14	0.22	0.24	0.163
**Variable**	**EB1**	**EB2**	**EB3**	**EB4**	**EB5**	**EB6**	**EB7**	**EB8**	**EB9**	***P*-value**		
**Categorical variables (expressed as odds ratios)**												
Sex**^a^**	0.88	**0**.**6**	0.9	1.2	1.1	0.8	0.9	1.2	1.3	0.27		
*APOE4* carriers	1.67	0.7	1.6	0.9	1.5	0.8	1.2	0.7	0.8	0.28		
Social disinhibition	**0**.**16**	**0**.**13**	**0**.**09**	1.95	**2**.**83**	1.48	0.95	1.07	2.07	**<0**.**001**		
Apathy	0.76	0.9	**0**.**6**	1.4	0.9	1.1	**0**.**6**	1.1	0.9	0.148		
Lack of empathy	**0**.**38**	**0**.**06**	**0**.**16**	1.26	1.98	0.39	0.85	0.92	0.86	**0**.**004**		
Perseverative/obsessive behaviour	**0**.**28**	**0**.**22**	0.46	1.39	**2**.**17**	0.50	1.10	0.51	0.96	**0**.**002**		
Hyperorality/dietary habit changes	**0**.**43**	**0**.**39**	**0**.**30**	1.16	1.51	1.61	1.56	1.05	1.35	**<0**.**001**		

Bold values are statistically significant. DR = delayed recall; EB = eigenbrain; IR = immediate recall; STMS = Short Test Of Mental Status; WCST = Wisconsin Card Sorting Test.

^a^An odds ratio >1 is associated with a higher likelihood of being female, whereas an odds ratio <1 is associated with a higher likelihood of being male.

Eigenbrain 1 (‘behavioural eigenbrain’) reflected a gradient of macro-scale cortical organization opposing frontotemporal areas to parieto-occipital areas bilaterally. Behavioural Alzheimer’s disease patients had significantly higher eigenvalues than other groups, meaning that these patients had higher hypometabolism in frontotemporal areas relative to parieto-occipital compared with other groups. Higher eigenvalues related with poorer verbal fluency performance and higher educational attainment, and a higher likelihood of presenting with behavioural/personality symptoms except apathy. Meta-analytic decoding revealed an association between higher eigenvalues and topics of moral and social reasoning, stimulus response, language perception, error learning and working memory, and between lower eigenvalues and topics of negative emotion, motion perception and numerical operations.

Eigenbrain 2 (‘cognitive eigenbrain’) opposed heteromodal cortices to unimodal, sensorimotor, orbitofrontal, ventromedial prefrontal and temporopolar areas bilaterally. Dysexecutive Alzheimer’s disease patients had significantly higher eigenvalues compared with all other groups, meaning that they exhibited relatively more hypometabolism in heteromodal cortices relative to unimodal, orbitofrontal, ventromedial prefrontal and temporopolar areas compared with other groups. Higher eigenvalues related to earlier age at symptom onset and worse performance on all cognitive tasks except for the WCST and measures of episodic memory (delayed recall), and a lower likelihood of presenting with behavioural/personality symptoms except apathy. Meta-analytic decoding revealed associations between higher eigenvalues and topics of response inhibition, hearing and language semantics, and between lower eigenvalues and topics of decision-making, error learning, mental imagery, language perception and memory.

Eigenbrain 3 (‘perception eigenbrain’) opposed frontal areas to temporo-parietal areas bilaterally. Dysexecutive and amnestic Alzheimer’s disease patients had higher eigenvalues compared with bvFTD. This means that dysexecutive and amnestic Alzheimer’s disease patients had more hypometabolism in temporo-parietal areas relative to frontal areas compared with bvFTD patients. Higher eigenvalues related to worse performance on the STMS and tasks requiring the processing of visual information (TMT-B, visuoconstruction, visual episodic memory), and a lower likelihood of presenting with behavioural/personality symptoms except perseverative behaviours. Meta-analytic decoding revealed associations between higher eigenvalues and topics of perception, mental imagery, negative emotion and visual attention, and between lower eigenvalues and topics of stimulus response, memory, motor, moral and social reasoning, numerical operations and language perception.

### Eigenbrain-based linear discriminant analysis

**Figure 4:**
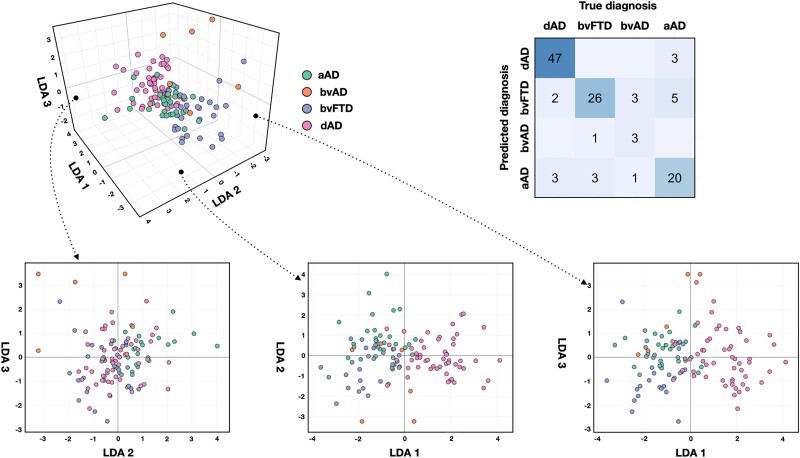
**Linear discriminant analysis embedding based on FDG-PET eigenbrains**. Three-dimensional embedding of patients based on the three linear discriminants resulting from the linear discriminant analysis (LDA) along with the confusion matrix showing true versus predicted diagnosis. aAD = amnestic Alzheimer’s disease; bvAD = behavioural Alzheimer’s disease; bvFTD = behavioural variant of frontotemporal dementia; dAD = dysexecutive Alzheimer’s disease; FDG = fluorodeoxyglucose.

The linear discriminant analysis yielded a solution of three linear discriminants. The 3D embedding of patients is displayed in Fig. 4 along with the confusion matrix between true and predicted diagnosis. The analysis achieved an accuracy of 82.1% in predicting diagnosis (90.4% for dysexecutive Alzheimer’s disease, 86.7% for bvFTD, 42.9% for behavioural Alzheimer’s disease, 71.4% for amnestic Alzheimer’s disease). This analysis supports that the latent factors, which were defined solely using biologically-relevant information from FDG-PET images regardless of phenotypic classes, do reflect an unbiased characterization that still captures clinical syndromic differences in brain metabolism across the studied patient cohort. A path towards a more generalizable description of latent factors related to the full spectrum of degenerative functional anatomy observed in clinical practice was recently described using a ‘global functional state space’ framework.^28^

Repeating this analysis using log-transformed eigenvalues as input features or a robust linear discriminant analysis yielded similar results in predicting diagnosis (80% and 82.9%, respectively), indicating that the slight heteroscedasticity observed had marginal influence on the results. Associations between the three linear discriminant analyses and age were significant, but a visual inspection of the data distribution did not reveal clear patterns above and beyond phenotypic differences. We nonetheless conducted a follow-up linear discriminant analysis using only age as an input feature, which yielded an accuracy of 67.5% in predicting diagnosis. This argues against the fact that mere age-related differences drove the results of the initial analysis. Mapping sex onto the low-dimensional space from the initial analysis did not reveal any coherent pattern. This supplementary analysis is displayed in Supplementary Fig. 5.

FDG-PET images of the patients misclassified by the linear discriminant analysis can be found in Supplementary Fig. 6. Briefly, cases misclassified as amnestic Alzheimer’s disease generally had mild, patchy patterns of hypometabolism involving posterior areas, cases predicted as bvFTD/behavioural Alzheimer’s disease generally had more frontotemporal hypometabolism including two amnestic Alzheimer’s disease cases predicted to be bvFTD with prominent temporopolar hypometabolism, and cases predicted as dysexecutive Alzheimer’s disease had more posterior involvement, including the posterior portion of the temporal lobe and parietal areas. This suggests that the linear discriminant analysis could accurately identify archetypical global patterns associated with the studied phenotypes and that these patients had particular, atypical imaging features that are not traditionally observed in their respective clinical syndromic classes.

## Discussion

Our results provide compelling evidence for distinct macro-scale patterns of degeneration underlying predominant dysexecutive versus behaviour/personality symptomatology in neurodegenerative diseases. Both traditional group-wise comparisons with clinical diagnosis as a starting point and machine learning approaches unbiased by clinical diagnosis highlight a correspondence between the degeneration of heteromodal cortices and worse cognitive symptomatology with a strong involvement of executive functions. This was supported by both the within-cohort brain-behaviour relationships and associations between eigenbrains and Neurosynth meta-analytic topics. These same analyses highlighted strong associations between relative hypometabolism in frontotemporal regions and a greater likelihood of presenting with behavioural/personality problems and meta-analytic topics such as moral reasoning and decision making, but lack of association with most cognitive measurements. Importantly, the eigenbrains yielded by the spectral decomposition of FDG-PET images reflect a relative distribution of metabolism opposing two sets of brain areas, which is equally mirrored in the brain-behaviour relationships expressed by these eigenbrains. Hence, patterns of hypometabolism strongly associated with cognitive symptomatology were also linked to a lower likelihood of presenting behavioural/personality problems and vice versa. It is also important to point out that the eigenbrains were derived using FDG-PET images across the whole patient sample, and therefore the brain-behaviour relationships reflected by these eigenbrains cover the syndromic diversity across the whole cohort and do not merely recapitulate features of distinct clinical phenotypes in isolation. Moreover, comparable disease duration across groups suggests that results were not biased by differences in disease progression. These patterns have been reported in other populations and with different imaging techniques (e.g. typical Alzheimer’s disease spectrum, normal ageing, young adults),^28,60,62^ which further supports their reproducibility. These findings provide a better understanding of the system-level pathophysiology of predominant neurodegenerative dysexecutive and behavioural phenotypes and are important for the development of criteria to diagnose and distinguish these syndromes. They also represent a considerable extension of our previous work characterizing the clinico-radiological features of dysexecutive Alzheimer’s disease^7^ and provide support for the global functional state space ontology as an operational framework to offer an unbiased data-driven classification degenerative disorders of mental functions.^28^

We demonstrate clear differences in patterns of degeneration distinguishing predominant dysexecutive versus behavioural dementia syndromes. For instance, relative hypometabolism in heteromodal cortices (eigenbrain 2) distinguished dysexecutive Alzheimer’s disease from all other phenotypes and exhibited associations with dysexecutive impairment and early age at disease onset. This is particularly interesting given that bvFTD and behavioural Alzheimer’s disease patients also presented, on average, with an early age at symptom onset, yet were not particularly represented by this pattern. This suggests that the pattern of hypometabolism represented by this eigenbrain reflects the interaction between ageing- and phenotype-related physiology rather than age-related effects alone. Relative hypometabolism in parieto-temporal areas (eigenbrain 3) distinguished dysexecutive and amnestic Alzheimer’s disease from bvFTD. Importantly, eigenbrains 2 and 3 related to impairment across a wide range of cognitive domains, especially executive functions, and a lower likelihood of manifesting behavioural/personality symptoms. This agrees with the literature suggesting that this principal gradient of macro-scale organization is thought to support the abstract modelling of internal and external data to guide complex behaviour and mental representations.^28,60^ In contrast, relative hypometabolism in frontotemporal areas (eigenbrains 1 and 3), distinguished both behavioural Alzheimer’s disease and bvFTD from dysexecutive and amnestic Alzheimer’s disease and was associated with a higher likelihood of manifesting behaviour/personality symptoms but milder cognitive impairment. These brain-behaviour relationships are in line with evidence showing that this set of regions would be important hubs for personality, social behaviour, and emotional regulation.^16,17^ Of note, the fact that eigenbrain 1 was not as distinctive of bvFTD compared with other phenotypes may be explained by the inclusion of cases with the right temporal variant of frontotemporal dementia^15^ that have been labelled as bvFTD, which may be better captured by the negative polarity of eigenbrain 2. The linear discriminant analysis discriminated perfectly between dysexecutive and behavioural Alzheimer’s disease. Additionally, no bvFTD patient was predicted to have dysexecutive Alzheimer’s disease, and only 2/52 dysexecutive Alzheimer’s patients were predicted to have bvFTD. In contrast, predictions for the behavioural Alzheimer’s disease group had the lowest accuracy, where nearly half of these patients were predicted to have bvFTD. This was expected given the large overlap in clinical features and patterns of FDG-PET hypometabolism between these syndromes. Taken together, these findings a double dissociation in patterns of macro-scale degeneration associated with predominant dysexecutive versus behavioural/personality symptomatology.^28^

Our findings call for a distinction between dysexecutive and behavioural degenerative dementia syndromes rather than considering them along the same phenotypic spectrum. In fact, they appear to exist on orthogonal spectrums as reflected in our decompositions of brain metabolism. This has implications for efforts to develop and implement clinical and research criteria for these syndromes. For instance, the recent research criteria for behavioural Alzheimer’s disease mandates the presence of episodic memory and/or executive functions impairment.^26^ While predominant behavioural syndromes can present with significant cognitive impairment, these latter symptoms are not at the forefront of the clinical picture and do not share the same macro-scale anatomy with predominant cognitive symptomatology, as per our results. We argue that criteria should place a greater emphasis on behavioural rather than cognitive symptoms. For instance, cognitive impairment could be included as a supportive feature or as a non-mandatory symptom that can be combined with initial and predominant behavioural symptoms, similarly to the established criteria for bvFTD.^2^ This would prevent the inclusion of patients with an initial and predominant dyscognitive syndrome who also present with or later develop mild behavioural symptomatology, as often seen in dysexecutive^1^ and amnestic Alzheimer’s disease.^63^ Our findings also highlight the importance of incorporating biomarkers to support the differential diagnosis of dysexecutive and behavioural syndromes, given that they can both result from different underlying pathologies despite sharing similar imaging and clinical features. This was demonstrated for behavioural Alzheimer’s disease and bvFTD in the present study but it is also the case for dysexecutive syndromes.^64,65^ We believe these implications are critical for the development of therapeutic strategies aimed at macro-scale network physiology, for instance repetitive magnetic stimulation of targeted functional networks,^66^ and the optimization of clinical trials including the enrollment of patients with atypical presentations and the definition of desired clinical endpoints.^21^

Some limitations must be considered. The sample size is modest, particularly for the behavioural Alzheimer’s disease group. However, the data-driven nature of our approach is independent from clinical diagnosis and leverages physiologically relevant brain data across the whole cohort, thus reducing the impact of the small sample size. Our study was retrospective and included a mixture of patients recruited from clinical practice and research. A consequence is that neurological and neuropsychological assessments were not standardized across settings. Moreover, while the inclusion of clinical and research data favors generalization, patients included in this study were recruited in a tertiary clinic, where atypical degenerative dementia phenotypes are more frequently encountered than in other settings. However, it is important to note that early-onset dementias are often misdiagnosed in non-specialized settings,^24,67^ and we hope our findings will translate into a better recognition of these disorders across clinical settings. It is also relevant to discuss potential circularity concerns between the use of FDG-PET imaging for diagnostic classification and the brain-behaviour relationships expressed by the eigenbrains. All current diagnostic schemas in clinical practice and research studies are inherently clinicopathologic entities, and neuroimaging is a key indicator of pathology that is commonly used in clinical practice. This can raise problems of circularity as it with all clinicopathologic elements commonly used in diagnosis. However, we wish to point out that FDG-PET imaging was used for diagnostic purposes in less than half of the patient sample, and the use of unsupervised data analysis techniques to identify the eigenbrains was independent of diagnostic status, thus mitigating concerns regarding circularity. Moreover, the patterns identified in the present study have also been found in normal ageing and across the Alzheimer’s disease spectrum in independent cohorts,^28^ which supports the reproducibility of these patterns. The relationship between these eigenbrains and cognitive symptoms in the same patients may similarly be confounded by circularity, but the Neurosynth analysis and a large established literature^21,28^ on these functional anatomic relationships also mitigate these concerns. It was also not possible to include an independent cohort to replicate our results, given the rarity of these phenotypes and the lack of FDG-PET in cohorts of individuals with early-onset dementia. However, the comparisons with meta-analytic topics from a large external database of neuroimaging studies supports the generalization and interpretability of our findings. Autopsy data was not available across the whole cohort, and thus it was not possible to assess how co-pathologies may have influenced the composition of the eigenbrains and clinico-radiological associations. Related to this point, although behavioural Alzheimer’s disease patients had evidence of Alzheimer’s pathology, autopsy data was missing for three of them. Hence this does not rule out the possibility of co-existing frontotemporal lobar degeneration pathology that may have contributed to the symptoms in these patients.

We leveraged traditional comparisons and machine learning approaches to uncover the macro-scale anatomy of neurodegenerative dementia syndromes primarily targeting dysexecutive versus behavioural/personality phenotypes. Our results strongly suggest a double dissociation in the macro-scale pathophysiology underlying the predominant symptomatology in these syndromes and call for a distinction between these phenotypes rather than considering them along the same spectrum. Our findings highlight the role of unbiased, data-driven techniques to inform the classification of neurodegenerative diseases. They also have critical implications for the development of clinical criteria to diagnose and distinguish these syndromes, patient care, and therapeutic strategies for atypical presentations of early-onset dementias.

## Supplementary Material

awad356_Supplementary_Data

## Data Availability

Data from the Mayo Clinic Study of Aging and the Mayo Clinic Alzheimer’s Disease Research Center are available upon request from these studies (https://www.mayo.edu/research/centers-programs/alzheimers-disease-research-center/data-requests). The MCALT can be found at: https://www.nitrc.org/projects/mcalt/.

## References

[1] Townley RA , Graff-Radford J, Mantyh WG, et al Progressive dysexecutive syndrome due to Alzheimer’s disease: A description of 55 cases and comparison to other phenotypes. Brain Commun. 2020;2:fcaa068. 32671341 10.1093/braincomms/fcaa068 PMC7325839

[2] Rascovsky K , Hodges JR, Knopman D, et al Sensitivity of revised diagnostic criteria for the behavioural variant of frontotemporal dementia. Brain. 2011;134:2456–2477. 21810890 10.1093/brain/awr179 PMC3170532

[3] Boeve BF , Boxer AL, Kumfor F, Pijnenburg Y, Rohrer JD. Advances and controversies in frontotemporal dementia: Diagnosis, biomarkers, and therapeutic considerations. Lancet Neurol. 2022;21:258–272. 35182511 10.1016/S1474-4422(21)00341-0

[4] Johnson JK , Vogt BA, Kim R, Cotman CW, Head E. Isolated executive impairment and associated frontal neuropathology. Dement Geriatr Cogn Disord. 2004;17:360–367. 15178954 10.1159/000078183 PMC2637356

[5] Smirnov DS , Salmon DP, Galasko D, et al Association of neurofibrillary tangle distribution with age at onset–related clinical heterogeneity in Alzheimer disease: An autopsy study. Neurology. 2022;98:e506–e517. 34810247 10.1212/WNL.0000000000013107 PMC8826459

[6] Johnson JK , Head E, Kim R, Starr A, Cotman CW. Clinical and pathological evidence for a frontal variant of Alzheimer disease. Arch Neurol. 1999;56:1233–1239. 10520939 10.1001/archneur.56.10.1233

[7] Corriveau-Lecavalier N , Barnard LR, Lee J, et al Deciphering the clinico-radiological heterogeneity of dysexecutive Alzheimer’s disease. Cereb Cortex. 2023;33:7026–7043. 36721911 10.1093/cercor/bhad017 PMC10233237

[8] Yeager BE , Bruss J, Duffau H, et al Central precuneus lesions are associated with impaired executive function. Brain Struct Funct. 2022;227:3099–3108. 36087124 10.1007/s00429-022-02556-0 PMC9743014

[9] Corriveau-Lecavalier N , Machulda MM, Botha H, et al Phenotypic subtypes of progressive dysexecutive syndrome due to Alzheimer’s disease: A series of clinical cases. J Neurol. 2022;269:4110–4128. 35211780 10.1007/s00415-022-11025-x PMC9308626

[10] Uddin LQ . Cognitive and behavioural flexibility: Neural mechanisms and clinical considerations. Nat Rev Neurosci. 2021;22:167–179. 33536614 10.1038/s41583-021-00428-w PMC7856857

[11] Selemon LD , Goldman-Rakic PS. Common cortical and subcortical targets of the dorsolateral prefrontal and posterior parietal cortices in the rhesus monkey: Evidence for a distributed neural network subserving spatially guided behavior. J Neurosci. 1988;8:4049–4068. 2846794 10.1523/JNEUROSCI.08-11-04049.1988 PMC6569486

[12] Goldman-Rakic PS . Topography of cognition: Parallel distributed networks in primate association cortex. Annu Rev Neurosci. 1988;11:137–156. 3284439 10.1146/annurev.ne.11.030188.001033

[13] Corriveau-Lecavalier N , Gunter JL, Kamykowski M, et al Default mode network failure and neurodegeneration across aging and amnestic and dysexecutive Alzheimer’s disease. Brain Commun. 2023;5:fcad058. 37013176 10.1093/braincomms/fcad058 PMC10066575

[14] Mesulam MM . Temporopolar regions of the human brain. Brain. 2023;146:20–41. 36331542 10.1093/brain/awac339 PMC10060717

[15] Ulugut Erkoyun H , Groot C, Heilbron R, et al A clinical-radiological framework of the right temporal variant of frontotemporal dementia. Brain. 2020;143:2831–2843. 32830218 10.1093/brain/awaa225 PMC9172625

[16] Ranasinghe KG , Rankin KP, Pressman PS, et al Distinct subtypes of behavioral variant frontotemporal dementia based on patterns of network degeneration. JAMA Neurol. 2021;73:1078–1088. 10.1001/jamaneurol.2016.2016 PMC5024785 27429218

[17] Jones DT , Graff-Radford J. Executive dysfunction and the prefrontal Cortex. Contin Lifelong Learn Neurol. 2021;27:1586–1601. 10.1212/CON.0000000000001009 34881727

[18] Possin KL , Feigenbaum D, Rankin KP, et al Dissociable executive functions in behavioral variant frontotemporal and Alzheimer dementias. Neurology. 2013;80:2180–2185. 23658382 10.1212/WNL.0b013e318296e940 PMC3721104

[19] Moura MVB , Mariano LI, Teixeira AL, Caramelli P, de Souza LC. Social cognition tests can discriminate behavioral variant frontotemporal dementia from Alzheimer’s disease independently of executive functioning. Arch Clin Neuropsychol. 2021;36:831–837. 33034347 10.1093/arclin/acaa084

[20] Gossink F , Schouws S, Krudop W, et al Social cognition differentiates behavioral variant frontotemporal dementia from other neurodegenerative diseases and psychiatric disorders. Am J Geriatr Psychiatry. 2018;26:569–579. 29501411 10.1016/j.jagp.2017.12.008

[21] Graff-radford J , Yong KXX, Apostolova LG, et al New insights into atypical Alzheimer’s disease in the era of biomarkers. Lancet Neurol. 2021;20:222–234. 33609479 10.1016/S1474-4422(20)30440-3 PMC8056394

[22] Bang J , Spina S, Miller BL. Frontotemporal dementia. Lancet. 2015;386:1672–1682. 26595641 10.1016/S0140-6736(15)00461-4 PMC5970949

[23] Mendez MF , Joshi A, Tassniyom K, Teng E, Shapira JS. Clinicopathologic differences among patients with behavioral variant frontotemporal dementia. Neurology. 2013;80:561–568. 23325909 10.1212/WNL.0b013e3182815547 PMC3589292

[24] Hendriks S , Peetoom K, Bakker C, et al Global prevalence of young-onset dementia: A systematic review and meta-analysis. JAMA Neurol. 2021;78:1080–1090. 34279544 10.1001/jamaneurol.2021.2161 PMC8290331

[25] Ossenkoppele R , Pijnenburg YAL, Perry DC, et al The behavioural/dysexecutive variant of Alzheimer’s disease: Clinical, neuroimaging and pathological features. Brain. 2015;138:2732–2749. 26141491 10.1093/brain/awv191 PMC4623840

[26] Ossenkoppele R , Singleton EH, Groot C, et al Research criteria for the behavioral variant of Alzheimer disease: A systematic review and meta-analysis. JAMA Neurol. 2021;78:961. 34870696 10.1001/jamaneurol.2021.4417 PMC8649917

[27] Dubois B , Villain N, Frisoni GB, et al Clinical diagnosis of Alzheimer’s disease: Recommendations of the international working group. Lancet Neurol. 2021;20:484–496. 33933186 10.1016/S1474-4422(21)00066-1 PMC8339877

[28] Jones D , Lowe V, Graff-Radford J, et al A computational model of neurodegeneration in Alzheimer’s disease. Nat Commun. 2022;13:1643. 35347127 10.1038/s41467-022-29047-4 PMC8960876

[29] McKhann GM , Knopman DS, Chertkow H, et al The diagnosis of dementia due to Alzheimer’s disease: Recommendations from the National Institute on Aging-Alzheimer’s Association workgroups on diagnostic guidelines for Alzheimer’s disease. Alzheimer’s Dement. 2011;7:263–269. 21514250 10.1016/j.jalz.2011.03.005 PMC3312024

[30] Beekly DL , Ramos EM, Lee WW, et al The National Alzheimer’s Coordinating Center (NACC) database: The uniform data set. Alzheimer Dis Assoc Disord. 2007;21:249–258. 17804958 10.1097/WAD.0b013e318142774e

[31] Roberts RO , Geda YE, Knopman DS, et al The Mayo Clinic study of aging: Design and sampling, participation, baseline measures and sample characteristics. Neuroepidemiology. 2008;30:58–69. 18259084 10.1159/000115751 PMC2821441

[32] Mirra SS , Heyman A, McKeel D, et al The consortium to establish a registry for Alzheimer’s disease (CERAD): Part II. Standardization of the neuropathologic assessment of Alzheimer’s disease. Neurology. 1991;41:479. 2011243 10.1212/wnl.41.4.479

[33] Hyman BT , Phelps CH, Beach TG, et al National institute on aging–Alzheimer’s association guidelines for the neuropathologic assessment of Alzheimer’s disease. Alzheimer’s Dement. 2012;8:1–13. 22265587 10.1016/j.jalz.2011.10.007 PMC3266529

[34] Braak H , Alafuzoff I, Arzberger T, Kretzschmar H, Del Tredici K. Staging of Alzheimer disease-associated neurofibrillary pathology using paraffin sections and immunocytochemistry. Acta Neuropathol. 2006;112:389–404. 16906426 10.1007/s00401-006-0127-z PMC3906709

[35] Dickson DW , Bergeron C, Chin SS, et al Office of rare diseases neuropathologic criteria for corticobasal degeneration. J Neuropathol Exp Neurol. 2002;61:935–946. 12430710 10.1093/jnen/61.11.935

[36] Montine TJ , Phelps CH, Beach TG, et al National institute on aging–Alzheimer’s association guidelines for the neuropathologic assessment of Alzheimer’s disease: A practical approach. Acta Neuropathol. 2012;123:1–11. 22101365 10.1007/s00401-011-0910-3 PMC3268003

[37] Mackenzie IRA , Neumann M, Baborie A, et al A harmonized classification system for FTLD-TDP pathology. Acta Neuropathol. 2011;122:111–113. 21644037 10.1007/s00401-011-0845-8 PMC3285143

[38] Nelson PT , Dickson DW, Trojanowski JQ, et al Limbic-predominant age-related TDP-43 encephalopathy (LATE): Consensus working group report. Brain. 2019;142:1503–1527. 31039256 10.1093/brain/awz099 PMC6536849

[39] Ramos EM , Dokuru DR, Van Berlo V, et al Genetic screening of a large series of North American sporadic and familial frontotemporal dementia cases. Alzheimer’s Dement. 2020;16:118–130. 31914217 10.1002/alz.12011 PMC7199807

[40] Jack CR , Wiste HJ, Weigand SD, et al Defining imaging biomarker cut points for brain aging and Alzheimer’s disease. Alzheimer’s Dement. 2017;13:205–216. 27697430 10.1016/j.jalz.2016.08.005 PMC5344738

[41] Lowe VJ , Lundt ES, Albertson SM, et al Tau-positron emission tomography correlates with neuropathology findings. Alzheimer’s Dement. 2020;16:561–571. 31784374 10.1016/j.jalz.2019.09.079 PMC7067654

[42] Kokmen E , Smith GE, Petersen RC, Tangalos E, Ivnik RC. The short test of mental Status: Correlations with standardized psychometric testing. Arch Neurol. 1991;48:725–728. 1859300 10.1001/archneur.1991.00530190071018

[43] Lucas JA , Ivnik RJ, Smith GE, et al Mayo’s older Americans normative studies: Category fluency norms. J Clin Exp Neuropsychol. 1998;20:194–200. 9777473 10.1076/jcen.20.2.194.1173

[44] Steinberg BA , Bieliauskas LA, Smith GE, Ivnik RJ. Mayo’s older Americans normative studies: Age-and IQ-adjusted norms for the trail-making test, the Stroop test, and MAE controlled oral word association test. Clin Neuropsychol. 2005;19(3–4):329–377. 16120535 10.1080/13854040590945210

[45] Machulda MM , Ivnik RJ, Smith GE, et al Mayo’s older Americans normative studies: Visual form discrimination and copy trial of the Rey–Osterrieth complex figure. J Clin Exp Neuropsychol. 2007;29:377–384. 17497561 10.1080/13803390600726803

[46] Petersen RC , Smith G, Kokmen E, Ivnik RJ, Tangalos EG. Memory function in normal aging. Neurology. 1992;42:396. 1736173 10.1212/wnl.42.2.396

[47] Spreen O , Strauss E. A compendium of neuropsychological tests: Administration, norms and commentary. 2nd ed. Oxford University Press; 1991.

[48] Grant DA , Berg EA. WCST-Wisconsin Card Sorting Test. J Exp Psychol. 1993;38:404.

[49] Stroop JR . Studies of interference in serial verbal reactions. J Exp Psychol. 1935;18:643–662.

[50] Weschler D . Weschler memory scale-Revised: Psychol Corp; 1987.

[51] Wechsler D . Wechsler adult intelligence scale–Fourth edition (WAIS-IV). NCS Pearson; 2008.

[52] Wechsler D . WMS-T. Administration and scoring manual: Psychol Corp; 1997.

[53] Rey A . L’examen Clinique en Psychologie. 2nd ed. Universitaires de France; 1964.

[54] Tombaugh TN , Kozak J, Rees L. Normative data stratified by age and education for two measures of verbal fluency: FAS and animal naming. Arch Clin Neuropsychol. 1999;14:167–177. 14590600

[55] Osterrieth PA . Le test de copie d’une figure complexe; contribution a l’etude de la perception et de la memoire. Arch Psychol (Geneve). 1944;30:286–356.

[56] Townley RA , Botha H, Graff-Radford J, et al Posterior cortical atrophy phenotypic heterogeneity revealed by decoding 18F-FDG-PET. Brain Commun. 2021;3:fcab182. 34805993 10.1093/braincomms/fcab182 PMC8600283

[57] Horn JL . Factors in factor analysis. Psychometrika. 1965;30:179–185. 14306381 10.1007/BF02289447

[58] Rubin TN , Koyejo O, Gorgolewski KJ, Jones N, Poldrack RA, Yarkoni T. Decoding brain activity using a large-scale probabilistic functional-anatomical atlas of human cognition. PLoS Comput Biol. 2017;13:e1005649 29059185 10.1371/journal.pcbi.1005649 PMC5683652

[59] Poldrack RA , Mumford JA, Schonberg T, Kalar D, Barman B. Discovering relations between mind, brain, and mental disorders using topic mapping. PLoS Comput Biol. 2012;8:e1002707. 23071428 10.1371/journal.pcbi.1002707 PMC3469446

[60] Margulies DS , Ghosh SS, Goulas A, et al Situating the default-mode network along a principal gradient of macroscale cortical organization. Proc Natl Acad Sci U S A. 2016;113:12574–12579. 27791099 10.1073/pnas.1608282113 PMC5098630

[61] Shine JM , Breakspear M, Bell PT, et al Human cognition involves the dynamic integration of neural activity and neuromodulatory systems. Nat Neurosci. 2019; 22:289–296. 30664771 10.1038/s41593-018-0312-0

[62] Brown JA , Lee AJ, Pasquini L, Seeley WW. A dynamic gradient architecture generates brain activity states. Neuroimage. 2022;261:119526. 35914669 10.1016/j.neuroimage.2022.119526 PMC9585924

[63] Weintraub S , Wicklund AH, Salmon DP. The neuropsychological profile of Alzheimer disease. Cold Spring Harb Perspect Med. 2012;2:a006171. 22474609 10.1101/cshperspect.a006171 PMC3312395

[64] Jones DT . Multiple aetiologies of the progressive dysexecutive syndrome and the importance of biomarkers. Brain Commun. 2020;2:fcaa127. 33216830 10.1093/braincomms/fcaa127 PMC7660034

[65] Corriveau-Lecavalier N , Li W, Ramanan VK, Drubach DA, Day GS, Jones DT. Three cases of Creutzfeldt–Jakob disease presenting with a predominant dysexecutive syndrome. J Neurol. 2022;269:4222–4228. 35233692 10.1007/s00415-022-11045-7 PMC9516260

[66] Koch G , Casula EP, Bonnì S, et al Precuneus magnetic stimulation for Alzheimer’s disease: A randomized, sham-controlled trial. Brain. 2022;145:3776–3786. 36281767 10.1093/brain/awac285 PMC9679166

[67] Corriveau-Lecavalier N , Alden EC, Stricker NH, Machulda MM, Jones DT. Failed performance on the test of memory malingering and misdiagnosis in individuals with early-onset dysexecutive Alzheimer’s disease. Arch Clin Neuropsychol. 2022;37:1199–1207. 35435228 10.1093/arclin/acac016 PMC9396449

