# Supplementary material for: Uncovering the distinct macro-scale anatomy of dysexecutive and behavioural degenerative diseases
Source: Brain. 2023 Oct 13;147(4):1483–96. doi: 10.1093/brain/awad356 (PMC10994526; doi:10.1093/brain/awad356)
Supplement: awad356_Supplementary_Data [file awad356_supplementary_data.pdf]

Supplementary Table 1 Pathological findings

	dAD (n = 1)	bvFTD (n = 3)	bvAD (n = 4)	aAD (n = 7)
Age at death	54	69.00 (5.20)	75.00 (2.45)	84.43 (5.52)
Time from FDG-PET to death (months) (mean, standard deviation)	40	25.67 (9.71)	47.25 (29.74)	46.29 (25.99)
Thal phase				
0	0	1	0	0
1-2	0	2	0	0
3	0	0	0	2
4-5	1	0	4	5
Braak stage				
0-II	0	2	0	0
III	0	1	0	0
IV	0	0	1	0
V	0	0	3	1
VI	1	0	0	6
Neuritic amyloid plaques score				
0 (none)	0	Not specified	0	0
1 (sparse)	0	Not specified	0	0
2 (moderate)	0	Not specified	0	0
3 (frequent)	1	Not specified	4	7
4R tau corticobasal degeneration	0	3	0	0
FTLD TDP-43	0	1	0	0
TDP-43 LATE staging				
0 (none)	1	3	2	5
1 (amygdala)	0	0	2	1
2 (hippocampus)	0	0	0	1
3 (neocortex)	0	0	0	0
Hippocampal sclerosis	0	0	0	1
Lewy body disease stage				
0 (none)	1	2	4	4
1 (brainstem)	0	0	0	0
2 (limbic/transitional)	0	1	0	0
3 (neocortex)	0	0	0	1
4 (amygdala-predominant)	0	0	0	2
Vascular pathology severity				
Mild	0	0	2	2
Moderate to severe	1	1	1	3
Cerebral amyloid angiopathy severity				
Mild	0	1	0	5
Moderate to severe	1	1	3	1

Supplementary Table 2 Meta-analytic decoding of eigenbrains using the Neurosynth database

Summary term	Neurosynth topic term	EB1	EB2	EB3	EB4	EB5	EB6	EB7	EB8	EB9
Stimulus response	4_stimulus_time_repetition	-0.49	0.17	0.32	-0.05	-0.05	0.18	0.01	-0.05	0.05
Hearing	6_auditory_speech_temporal	0.20	-0.35	-0.19	0.12	0.18	-0.17	-0.09	0.03	-0.08
Reward	7_reward_feedback_striatum	0.08	0.02	0.00	0.17	0.13	0.03	-0.05	0.04	-0.32
Social	8_mpfc_social_medial	-0.37	0.15	0.22	-0.09	-0.16	0.31	0.11	-0.03	0.00
Working memory	9_memory_working_wm	-0.29	-0.15	0.01	0.04	0.28	0.06	0.03	0.22	0.09
Error learning	11_learning_training_practice	-0.39	0.25	0.08	-0.07	-0.08	0.22	0.05	-0.06	0.11
Response inhibition	16_response_inhibition_control	0.22	-0.34	0.09	-0.09	-0.12	-0.17	-0.08	-0.09	0.00
Motor	17_motor_cortex_hand	-0.02	-0.21	0.28	-0.05	-0.27	-0.14	0.01	-0.09	-0.07
Numerical	18_number_ips_numerical	0.40	0.09	0.19	-0.10	-0.19	-0.31	0.03	-0.14	-0.27
Negative emotion	26_emotional_amygdala_negative	0.53	-0.06	-0.24	0.04	-0.13	-0.23	-0.09	-0.24	-0.04
Moral	28_social_empathy_moral	-0.53	0.15	0.10	-0.07	-0.13	0.11	0.16	0.04	0.05
Decision making	30_decision_making_risk	-0.38	0.31	0.04	-0.06	-0.06	0.16	0.13	0.05	0.02
Pain	32_pain_somatosensory_stimulation	0.08	-0.12	0.02	-0.02	-0.11	0.00	-0.05	0.06	-0.03
Memory	33_memory_retrieval_encoding	-0.02	0.20	0.30	0.00	-0.17	-0.09	0.02	-0.10	-0.26
Language perception	37_language_reading_word	-0.44	0.20	0.17	-0.08	-0.20	0.13	0.15	0.00	0.01
Language semantics	38_semantic_category_representations	0.10	-0.24	-0.13	-0.04	0.37	0.02	-0.08	-0.01	-0.07
Facial recognition	40_face_faces_facial	0.19	0.14	0.10	-0.04	-0.19	-0.28	0.04	-0.06	-0.20
Mental imagery	41_imagery_mental_events	-0.01	0.23	-0.26	0.21	0.08	0.16	-0.06	0.03	0.31
Perception	42_visual_cortex_sensory	0.21	0.03	-0.26	0.04	0.12	0.09	-0.05	0.13	0.16
Directed gaze	44_eye_sleep_gaze	0.02	0.02	0.11	0.11	0.08	0.18	-0.05	0.10	-0.04
Motion perception	45_motion_perception_visual	0.44	0.07	-0.04	-0.05	-0.24	-0.13	-0.01	-0.11	-0.02
Visual attention	47_attention_attentional_target	0.12	0.05	-0.13	-0.02	0.14	-0.19	0.03	-0.04	-0.13

EB = Eigenbrain.

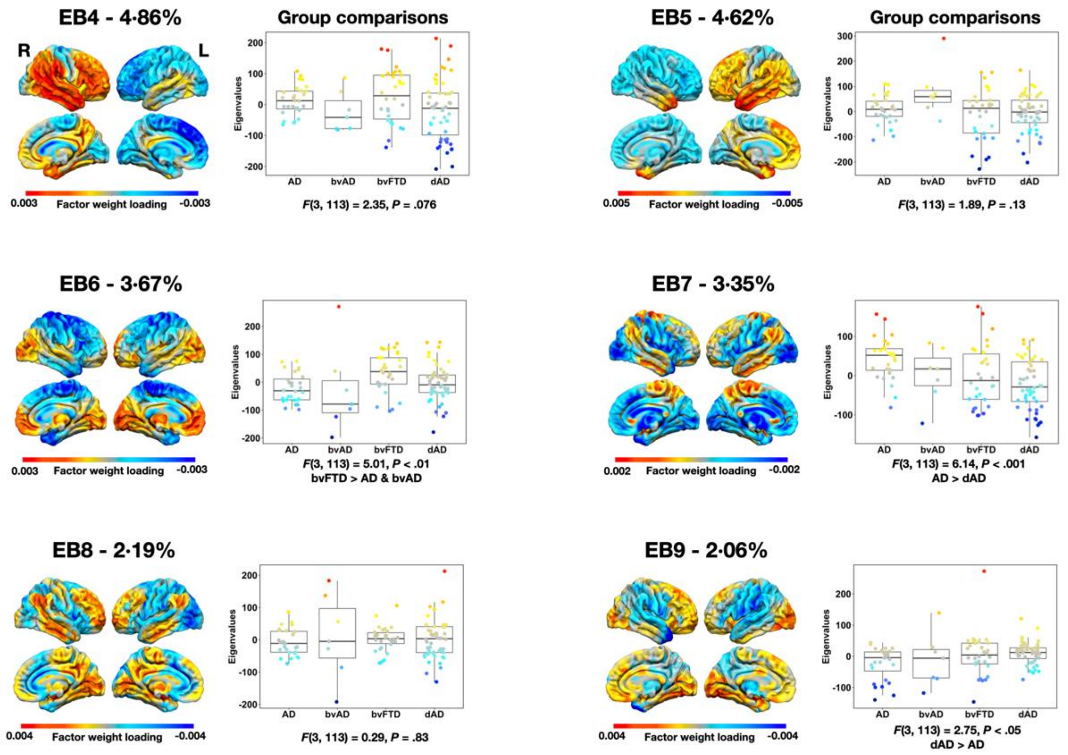

Supplementary Figure 1. Associations between eigenbrains 4-9 and diagnostic group. The color code indicates areas of relative FDG-PET hypometabolism highlighted by a given EB. These EBs reflect relative metabolism between two sets of brain areas, and the directionality (positive or negative) is arbitrary. The percentage of covariance explained between FDG-PET images is displayed above each EB rendering. EB = Eigenbrain; AD = Amnestic Alzheimer's disease; dAD = Dysexecutive AD; bvAD = Behavioral AD; bvFTD = Behavioral variant of fronto-temporal dementia.

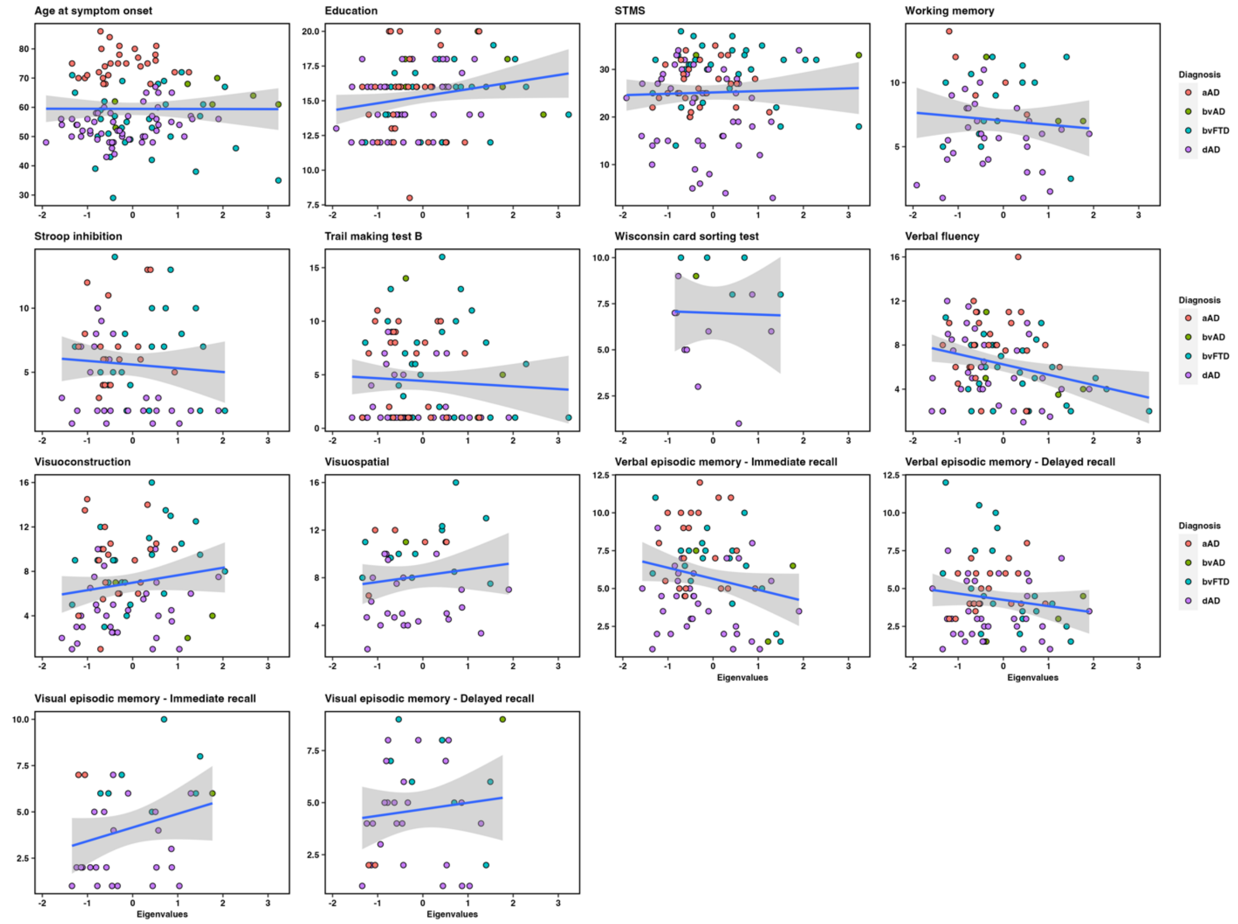

Supplementary Figure 2. Associations between eigenbrain 1 and demographic and cognitive variables. aAD = Amnestic Alzheimer's disease; dAD = Dysexecutive AD; bvAD = Behavioral AD; bvFTD = Behavioral variant of fronto-temporal dementia; STMS = Short test of mental status.

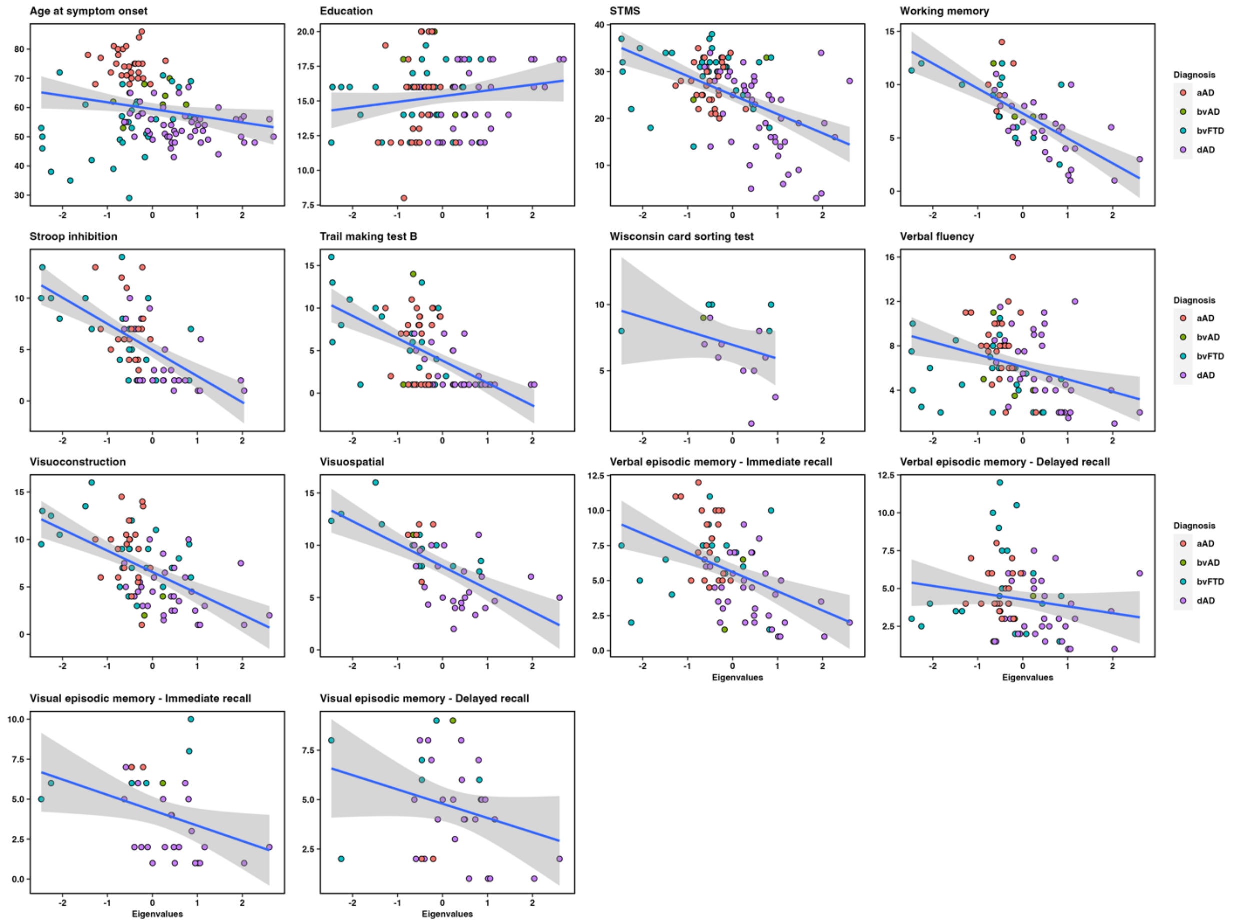

Supplementary Figure 3. Associations between eigenbrain 2 and demographic and cognitive variables. aAD = Amnestic Alzheimer's disease; dAD = Dysexecutive AD; bvAD = Behavioral AD; bvFTD = Behavioral variant of fronto-temporal dementia; STMS = Short test of mental status.

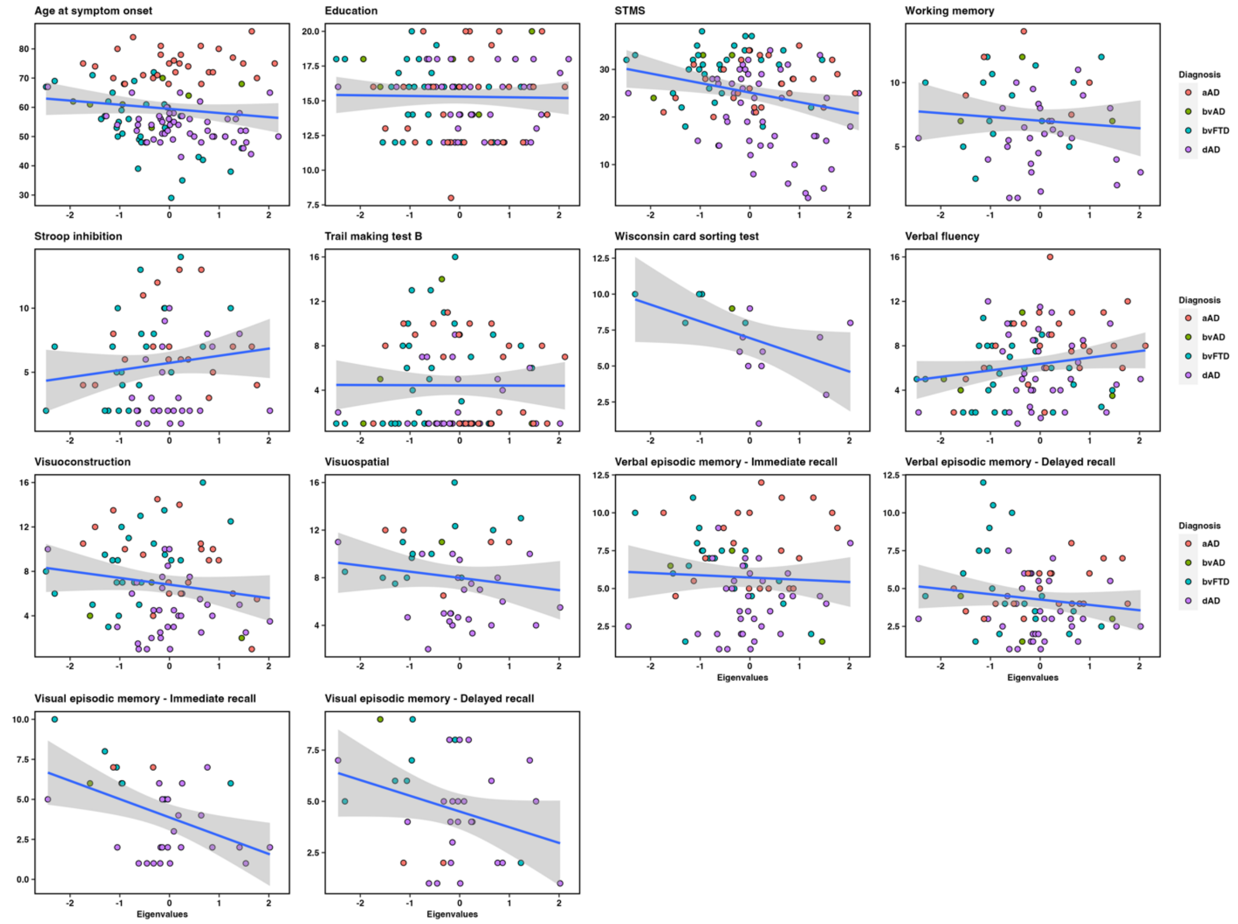

Supplementary Figure 4. Associations between eigenbrain 2 and demographic and cognitive variables. aAD = Amnestic Alzheimer's disease; dAD = Dysexecutive AD; bvAD = Behavioral AD; bvFTD = Behavioral variant of fronto-temporal dementia; STMS = Short test of mental status.

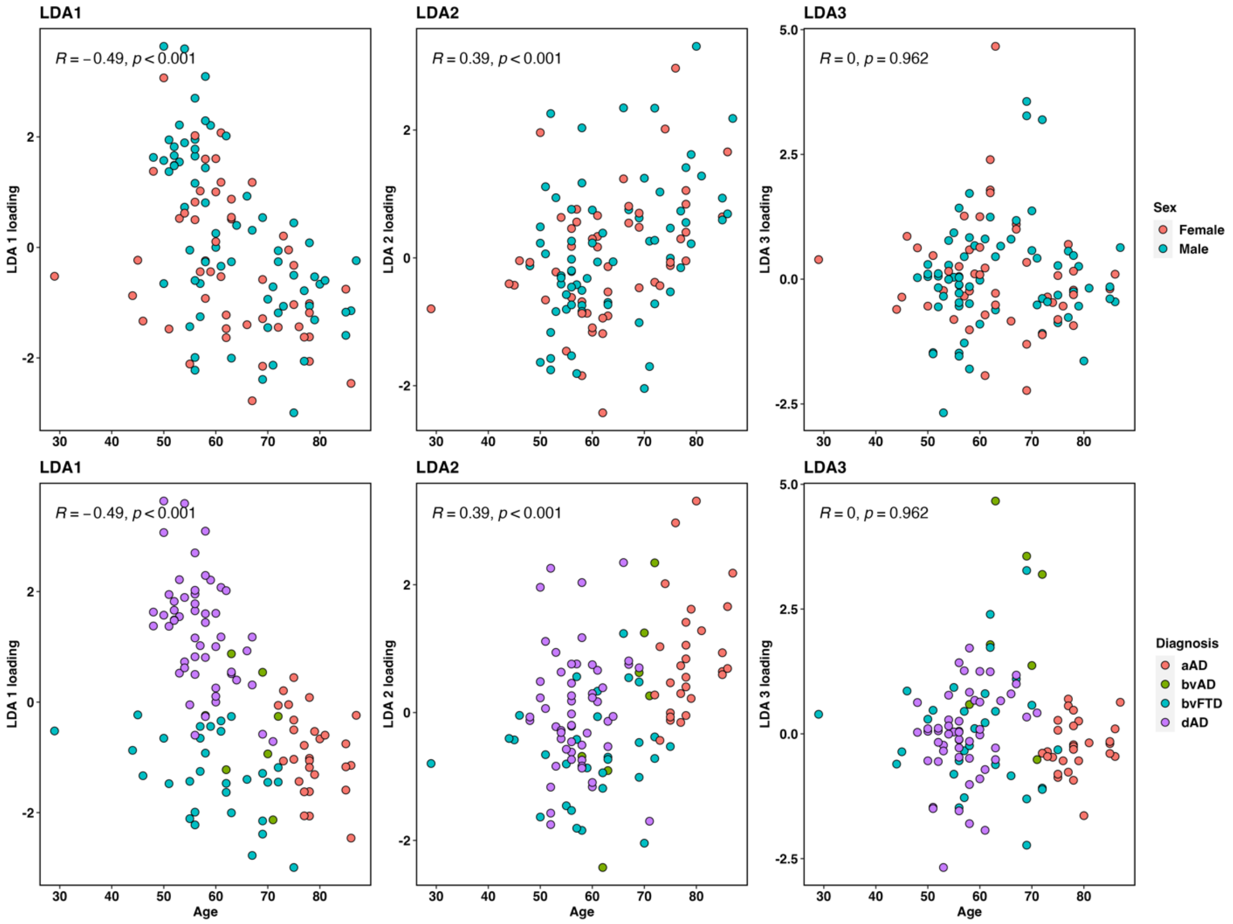

Supplementary Figure 5. *Associations between the linear discriminants and age and sex*. The correlation coefficients were generated using simple regression models. LDA = Linear discriminant analysis; aAD = amnestic Alzheimer’s disease; dAD = Dysexecutive AD; bvAD = Behavioral AD; bvFTD = Behavioral variant of fronto-temporal dementia.

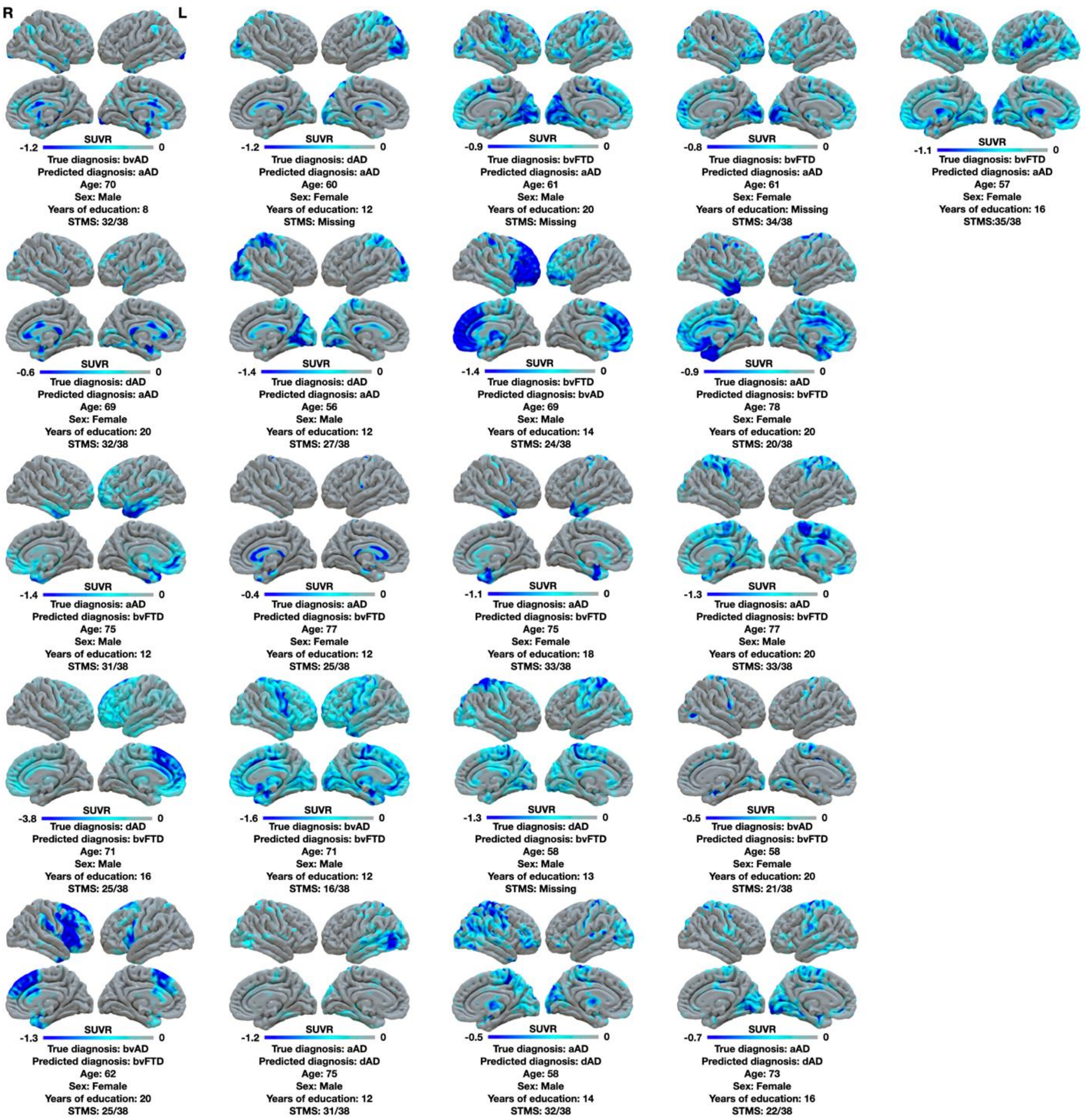

Supplementary Figure 6. FDG-PET images of patients misclassified by the linear discriminant analysis. The color scale represents SUVR values normalized to the pons. aAD = Amnestic Alzheimer's disease; dAD = Dysexecutive AD; bvAD = Behavioral AD; bvFTD = Behavioral variant of fronto-temporal dementia; STMS = Short test of mental status; SUVR = Standard uptake value ratio.
